# Molecular characterization of mismatch repair deficient tumors in young Jordanian patients

**DOI:** 10.1007/s12672-026-05717-3

**Published:** 2026-08-17

**Authors:** Olfat Ahmad, Iyad Sultan, Maysa Hussaini, Osama Alsmadi, Christian Sutter, Steffen Hirsch, Robert J. Autry, Christopher Previti, Asta Försti, Benjamin Schwalm, Lama AbuJamous, Nisreen Amayiri, Mayada Abu Shanap, Rola Amareen, Salsabeel Aljawabrah, Lara Fryjat, Aya Al-Naimat, Areen Qaisi, Hala Alnaimat, David E. Reuss, Nicholas R. Fernandez, Anirban Das, Uri Tabori, Stefan M. Pfister, Christian P. Schaaf

**Affiliations:** 1 Hopp Children’s Cancer Center (KiTZ), Heidelberg, Germany; 2 Division of Pediatric Neurooncology, German Cancer Research Center (DKFZ), German Cancer Consortium (DKTK), Heidelberg, Germany; 3 King Hussein Cancer Center (KHCC), Amman, Jordan; 4 Institute of Human Genetics, Heidelberg University, Im Neuenheimer Feld 366, 69120 Heidelberg, Germany; 5 Department of Pediatric Hematology and Oncology, Heidelberg University Hospital, Heidelberg, Germany; 6 Qatar University, Doha, Qatar; 7 Faculty of Medicine, University of Jordan, Amman, Jordan; 8 Department of Neuropathology, Institute of Pathology, University Hospital Heidelberg, Heidelberg, Germany; 9 Clinical Cooperation Unit Neuropathology, German Consortium for Translational Cancer Research (DKTK), German Cancer Research Center (DKFZ), Heidelberg, Germany; 10 Arthur and Sonia Labatt Brain Tumor Research Centre, Toronto, ON Canada; 11 National Center for Tumor Diseases (NCT) Heidelberg, Heidelberg, Germany

**Keywords:** Mismatch repair deficiency (MMRD), Constitutional mismatch repair deficiency (CMMRD), Lynch syndrome, Colorectal cancer, High-grade glioma, Jordan/Middle East

## Abstract

Mismatch repair deficiency (MMRD) contributes substantially to early-onset colorectal carcinoma (CRC) and pediatric malignancies in Jordan. Approximately 19% of CRCs diagnosed in adults younger than 45 years exhibit MMRD by immunohistochemistry (IHC). In pediatric tumors, MMRD accounts for 39% of high-grade gliomas (HGGs) and 44% of CRCs, likely reflecting the increased prevalence of constitutional MMRD (CMMRD) in this highly consanguineous population. However, the molecular characterization of MMRD-associated tumors in the Middle East remains limited. We analyzed 14 Jordanian patients (< 45 years) from 12 families with clinical or IHC evidence of MMRD, including four HGGs and 11 CRCs; five patients were children. Pathogenic or likely pathogenic *MMR* variants were identified in nine families, including one sibling pair, comprising five frameshift indels, three single nucleotide variants, and one large deletion. A variant of uncertain significance in *MSH6* was identified in another sibling pair. Apparent large deletions suggestive of sequencing artifacts in six patients highlighted the need for confirmatory copy number analysis by multiplex ligation-dependent probe amplification. Tumor mutational burden ranged from 2 to 352 variants/Mb (median, 23). Microsatellite instability was low in seven tumors, likely reflecting degraded DNA from older formalin-fixed paraffin-embedded samples. Mutational signatures were concordant with the molecular findings in most cases. Notably, the only ultrahypermutated tumor harbored a canonical somatic *POLE* p.(Val411Leu) proofreading-domain mutation together with polymerase-associated mutational signatures, consistent with combined MMRD and polymerase proofreading deficiency. This study represents the first comprehensive molecular characterization of MMRD-associated tumors in Jordan.

**Supplementary Information:**

The online version contains supplementary material available at 10.1007/s12672-026-05717-3.

## Introduction

Lynch syndrome (LS) is one of the most common adult-onset hereditary cancer predisposition syndromes (CPSs), caused by heterozygous pathogenic (P) or likely pathogenic (LP) variants in the DNA mismatch repair (*MMR*) genes *MLH1*, *MSH2*, *MSH6*, and *PMS2*, together with monoallelic deletions of the *EPCAM* gene vicinal to *MSH2* [1]. On the other hand, constitutional mismatch repair deficiency (CMMRD) syndrome, a more severe manifestation of MMR deficiency (MMRD) due to biallelic P/LP variants in the MMR genes, typically leads to pediatric onset of cancers [2]. Similar to global figures, the rate of suspected LS in young adult Jordanians with colorectal carcinoma (CRC) has been estimated to be 19% by immunohistochemistry (IHC) [3].

Notably, CMMRD is more commonly reported in the Near and Middle East compared to other regions, primarily due to higher rates of consanguinity, which increases the likelihood of inheriting biallelic MMR mutations [4, 5]. For example, while the consanguinity rate in Jordan is estimated to be around 30%, and up to 70% in other Middle and Near Eastern countries, it is less than 4% in most other regions worldwide [6]. Not surprisingly therefore, previous reports from Jordan showed high frequencies of pediatric MMRD CRC (44%) and high-grade glioma (HGG) (39%) [4, 5].

In both LS and CMMRD, the defective MMR system results in microsatellite instability (MSI) and increased predisposition to tumors with high tumor mutational burden (TMB). While there are some differences in the tumor spectrum caused by the two syndromes, both predispose mainly to CRC and brain tumors, among other malignancies. Despite the significant contribution of MMRD to CRC and HGG in Jordanian pediatric and young adult populations, molecular characterization of MMRD-associated tumors from Jordan and the broader Middle East remains limited [7].

In this case series, we aim to provide insights into molecular characterization of young Jordanian patients (< 45 years) with MMRD-associated HGG and/or CRC. For cases in which no pathogenic MMR variant was identified, an extended hereditary CPS gene panel was additionally analyzed.

## Materials and methods

### Patients cohort

This is a retrospective analysis of Jordanian patients conducted as a collaboration between King Hussein Cancer Center (KHCC), the German Cancer Research Center (DKFZ) and the Institute of Human Genetics Heidelberg, Germany. Patients younger than 45 years, clinically diagnosed with a biopsy-proven CRC or brain tumor at KHCC from January 1991 until October 2021 were recruited and shortlisted according to their MMR status as suggested by IHC of the corresponding four MMR proteins (MLH1, MSH2, MSH6 and PMS2), or if they scored ≥ 3 on CMMRD criteria regardless of staining pattern [8]. Institutional Review Board (IRB) approval from KHCC was obtained for contacting living patients, collecting their pedigrees, and obtaining blood samples for germline analysis at DKFZ. In addition, DNA was extracted from Formalin-Fixed Paraffin-Embedded (FFPE) sections and shipped to DKFZ for molecular charcterization.

### Pathology review

At KHCC, pathology reports and tumor blocks were reviewed for MMRD by IHC of the corresponding four MMR proteins (PMS2, MSH6, MLH1, and MSH2). Ventana Benchmark © automated immunostainer © was used to run the IHC stains according to the manufacturer’s instructions. Positive and negative controls were applied on each slide. A tumor was considered MMR proficient if nuclear staining of all of the four MMR proteins was detected in the studied tumor cells, regardless of the intensity of staining or the percentage of positive cell nuclei. MMR-deficient tumors exhibited an absence of any detectable nuclear signal in either one or two MMR proteins in the tumor cells by definition; the absence of nuclear MMR staining of both tumor and normal cells is a hallmark of CMMRD, while intact nuclear staining of normal cells suggested LS.

### Molecular analysis

Paired germline (leukocyte) and tumor DNA underwent whole-exome sequencing (WES) and low-coverage whole-genome sequencing (lcWGS) on a NovaSeq 6000 PE150 S4 platform (Illumina, San Diego, CA, USA). WES libraries were prepared using the SureSelectXT Automation Reagent Kit and SureSelectXT Human All Exon V7 Capture Library (Agilent Technologies) according to the manufacturer’s instructions, achieving a mean coverage of 63×–80×. lcWGS libraries were generated from the pre-capture libraries, with a mean coverage of 0.8×–1.2×. DNA methylation profiling was performed on tumor DNA using the Illumina Infinium MethylationEPIC array when indicated, as described below.

Germline WES data were aligned to the GRCh37/hg19 human reference genome using Biomedical Genomics Workbench (Qiagen, Hilden, Germany) and analyzed using Ingenuity Variant Analysis (Qiagen) together with the DKFZ in-house NGS analysis workflow for the identification of single nucleotide variants (SNVs), indels, and copy number variants (CNVs) affecting *MMR* genes.

CNVs were initially assessed from WES data using CNVkit v2.1.0 [9], which was used to identify candidate CNVs and generate B-allele frequency (BAF) plots. For the six patients with apparent MSH2 or MLH1 deletions identified by CNVkit, CNV profiles were subsequently generated from lcWGS data, and Multiplex Ligation-dependent Probe Amplification (MLPA) was performed for orthogonal validation. MLPA was carried out using the P003-D1 probe mix (MRC-Holland), which interrogates MLH1, MSH2, and EPCAM exon 9. Samples were first assessed for adequate hybridization and ligation using the three internal quality-control peaks. A heterozygous deletion of *MSH2* exon 7 was confirmed in one patient by a ~ 50% reduction in probe signal intensity. This finding was independently verified using a second MLPA kit (P248-B1) containing a probe located 51 nucleotides downstream of exon 7, which also enabled approximate delineation of the deletion boundaries. No deletions or duplications were detected by MLPA in the remaining patients.

Patients without an identifiable germline SNV, indel, or CNV in the MMR genes (*n* = 3), as well as those harboring a germline *MMR* variant of uncertain significance (*n* = 2), underwent additional analyses. Somatic *MMR* events and *BRAF* V600E status were assessed using the DKFZ in-house workflow, while *MLH1* promoter methylation was evaluated in RStudio (v4.2.0) by generating a heatmap using Illumina Infinium MethylationEPIC array data for four informative CpG sites within the *MLH1* promoter (cg23658326, cg11600697, cg21490561, and cg00893636) [10]. In addition, germline DNA from these five patients was analyzed using a cancer predisposition syndrome (CPS) panel comprising 213 genes (Supplementary Information.

To predict the pathogenicity/impairment of genetic variants, we utilized several computational tools, including SIFT (http://sift.jcvi.org/) and PolyPhen-2 (http://genetics.bwh.harvard.edu/pph2/). Additionally, we referred to Varsome (https://landing.varsome.com/varsome), ClinVar (https://www.ncbi.nlm.nih.gov/clinvar/), and gnomAD (https://gnomad.broadinstitute.org/) databases to gather further insights into the clinical significance and pathogenicity of the identified variants.

TMB was calculated based on WES data, as the number of high-quality (quality score ≥ 8) somatic functional SNVs and Indels in exonic regions divided by the total size of the exonic regions in the genome (total targeted regions overlapping annotated exons), multiplied by 1 million (TMB = mutations/genome × 1,000,000). Target regions were derived from the Agilent 7 (without Untranslated Region (UTRs)) exome enrichment kit, and annotations were based on Gencode version 19. Mutational signatures were generated utilizing paired normal/tumor WES data via COSMIC signature assignment tool https://cancer.sanger.ac.uk/signatures/assignment/. MSI was assessed on the basis of NGS data of WES utilizing MSIsensor https://github.com/ding-lab/msisensor [9]. We used a cut-off MSIsensor score ≥ 10 to define MSI high (MSI-H) [10].

## Results

### Clinical characteristics of the study cohort

Fourteen patients from 12 unrelated Jordanian families, all diagnosed before the age of 45 years, were enrolled in the study. The cohort comprised 15 tumors, including 11 CRCs and four HGGs, with one patient developing both an HGG at 22 years of age and a CRC two years later (patient #12, Table 1). Five patients were diagnosed during childhood, including two siblings with CRCs diagnosed at 13 and 15 years of age and three patients with HGGs diagnosed at 9, 13, and 17 years. Based on IHC, nine patients showed staining patterns consistent with LS, three demonstrated patterns suggestive of CMMRD, and two had retained MMR protein expression despite fulfilling the C4CMMRD clinical criteria, raising a strong suspicion of CMMRD according to the recommendations of the International Consensus Working Group [8].

**Table 1:** Clinical and molecular characterization of enrolled patients in the study

No.	Study ID	Age at diagnosis (year)	Sex	Tumor type	MSI (% Somatic sites)	TMB	IHCneg	Germline variant	COSMIC mutational signatures	Clinical criteria met	Conclusion diagnosis	Sequencing artifacts
1	140F4SD	39	M	CRC	H (14%)	49	MSH2 and MSH6 LS pattern	None (Candidate somatic structural inversion involving *MSH6* exon 2–intron 2 inversion: Chr2:48,018,253–48,018,370, supported by 3 reads).	SBS1 (0.32) SBS5(0.343) SBS6 (0.337)	Bethesda Guidelines	Suspected Somatic MMRD	*MSH2*, chr2:47,641,377 − 48,033,551(GRCh37), Lg Del (~ 390 kb, by rough computational mapping of potentially deleted regions identified by CNVkit)
2	140F12SD	30	F	CRC	H (16.35%)	38	MSH2 and MSH6 LS pattern	*MSH2* exon 7 deletion, heterozygous. Pathogenic	SBS1 (0.423) SBS5 (0.291) SBS6 (0.227)	Amsterdam II and Bethesda Guidelines	Lynch Syndrome Confirmed	*MSH2*, chr2:47,635,736 − 47,710,309(GRCh37), Lg Del(~ 74 kb, by rough computational mapping of potentially deleted regions identified by CNVkit)
3	140F24SD	44	M	CRC	H (21.95%)	42.73	PMS2 and MLH1 LS pattern	None (Candidate somatic *MLH1* inversion involving intron 12/exon 13: Chr3:37070267–37070403. Somatic, supported by 3–4 reads).	SBS5 (0.436) SBS6 (0.435)	Bethesda Guidelines	Suspected Somatic MMRD	*MLH1*, chr3:g.37,037,285 − 37,090,106(GRCh37), Lg Del(~ 53 kb, by rough computational mapping of potentially deleted regions identified by CNVkit)
4	140F2SD	33	M	CRC	NA	NA	MSH2 and MSH6 LS pattern	*MSH2*(NM_000251.3): c.1321dup p.Thr441fs. Heterozygous, AF = 0.53. Pathogenic	SBS1 (0.282) SBS5 (0.297) SBS6 (0.283)	Bethesda Guidelines	Lynch Syndrome Confirmed	–
5	140F30SD	28	M	CRC	L (5.8%)	25	PMS2, MLH1 LS pattern	*MLH1*(NM_000249.4): c.1164_1165insGAAT p.Ser388fs. Heterozygous, AF = 0.47. Pathogenic	SBS1 (0.15) SBS5 (0.539) SBS12 (0.251)	Amsterdam II and Bethesda Guidelines	Lynch Syndrome Confirmed	–
6	140F29S1D	13	F	CRC	L (8.75%)	19	PMS2 CMMRD pattern	*PMS2*(NM_000535.7): c.903G > T p.Lys301Asn. Homozygous, AF = 1. Likely pathogenic	SBS5 (0.356) SBS12 (0.268) SBS15 (0.162) SBS54 (0.169)	C4CMMRD	CMMRD Confirmed	–
7	140F29S2D	15	F	CRC	L (4.35%)	5	PMS2 CMMRD pattern	*PMS2*(NM_000535.7): c.903G > T p.Lys301Asn. Homozygous, AF = 1. Likely pathogenic	SBS5 (0.345) SBS15 (0.191) SBS26 (0.338)	C4CMMRD	CMMRD Confirmed	–
8	140F19SD1	37	M	CRC	H (18.52%)	40	PMS2, MLH1 and MSH6 LS pattern	*MLH1*(NM_000249.4):c.901del p.(Gln301ArgfsTer66). Heterzozygous, AF = 0.47. Pathogenic	SBS5 (0.409) SBS15 (0.129) SBS44 (0.281)	Amsterdam II and Bethesda Guidelines	CMMRD Confirmed	–
9	140F25SD	43	F	CRC	H (26.6%)	46	PMS2 and MLH1 LS pattern	*MLH1*(NM_000249.4): c.1836_1839del p.(Val612del). Heterozygous, AF = 0.48. Likely pathogenic	SBS5 (0.453) SBS6 (0.274) SBS54 (0.092)	Amsterdam II and Bethesda Guidelines	Lynch Syndrome Confirmed	–
10	140F20SD	38	M	CRC	L (0.13%)	2.1	MSH2 and MSH6 LS pattern	None (No candidate somatic MMR event identified either)	SBS5 (0.659) SBS1 (0.341)	Bethesda Guidelines	sporadic CRC of unresolved molecular etiology	*MSH2*, chr2:g.47635736_47748646del, Lg del (~ 112 kb, by rough computational mapping of potentially deleted regions identified by CNVkit).
11	140F46SD	17	F	HGG	L (1.5%)	15.3	MSH6 LS pattern	*MSH6*(NM_000179.3): c.2314 C > T p.(Arg772Trp) Homozygous, AF = 1 Pathogenic	SBS5 (0.233) SBS1 (0.767)	C4CMMRD	CMMRD Confirmed	chr3:g.37038204_37091970del, MLH1 exons 2–19, Lg del (~ 54 kb, by rough computational mapping of potentially deleted regions identified by CNVkit).
12	140F42S1D	22	M	HGG (22 years) Then CRC (24 years)	L (7%)	22.8	Retained IHC satins	*MSH6*(NM_000179.3): c.1217G > A p.(Cys406Tyr) Homozygous, AF = 1 VUS	SBS5 (0.332) SBS1 (0.355) SBS15 (0.202) SBS20 (0.112)	C4CMMRD	CMMRD Likely Diagnosis	None
13	140F42S2D	13	F	HGG	L (2%)	352	Retained IHC stains	*MSH6*(NM_000179.3): c.1217G > A p.(Cys406Tyr) Homozygous, AF = 1 VUS	SBS5 (0.368) SBS14 (0.103) SBS15 (0.296)	C4CMMRD	CMMRD Likely Diagnosis	*MLH1*, chr3:g.37038204_37170640del, intron 2–3’UTR, Lg del (~ 132 kb, by rough computational mapping of potentially deleted regions identified by CNVkit).
14	140F45SD	9	F	HGG	L (4.5%)	7.6	PMS2, CMMRD pattern	*PMS2*(NM_000535.7): c.1587dupG: p.(Gln530AlafsTer12), Homozygous, AF = 0.89 Pathogenic	SBS5 (0.346) SBS15 (0.305) SBS26 (0.325)	C4CMMRD	CMMRD Confirmed	–

No, Number; ID, Identifier; MSI, Microsatellite Instability; TMB, Tumor Mutational Burden; IHC, Immunohistochemistry; CRC, Colorectal Cancer; LS, Lynch Syndrome; M, Male; H, High; Chr, Chromosome; lg, Large; Del, Deletion; GRCh37, Genome Reference Consortium Human Build 37; NA, Not available; kb, Kilo base; c., Codon; p., Protein; Dup, Duplication; Fs, Frameshift; L, Low; UTR, Untranslated region; CMMRD, Constitutional Mismatch Repair Deficiency; IHCneg, Immunohistochemistry negative for mismatch repair; MMRP, Mismatch Repair Proficient

### Identified germline variants of *MMR* genes

Five distinct frameshift (fs) indels of *MMR* genes were identified in five unrelated patients, including 3 *MLH1* variants (patients # 5, 8 and 9), one *MSH2* variant (patient #4) and another *PMS2* variant (patient #14).

- Patient #4 (Table 1) is a 33-year-old male with CRC whose brother has also developed CRC. He carries *MSH2* variant NM_000251.3:c.1321dup (p.Thr441fs), which is fs variant classified as P variant in ClinVar.
- Patient # 5 (Table 1) is a 28-year-old male with CRC and a family history of various tumors including CRC, gastric, esophageal, endometrial, brain, and breast cancers. He has a fs variant in *MLH1* gene NM_000249.4:c.1164_1165insGAAT: p.(Ser388fs), which is classified as P variant in Clinvar.
- Patient #8 (Table 1) is a 37-year-old male with a family history notable for multiple relatives affected predominantly by CRC and two cases of breast cancer. He carries the *MLH1* variant NM_000249.4:c.901del: p.(Gln301ArgfsTer66), a fs alteration confirmed to be P variant in Clinvar.
- Patient #9 (Table 1) is a 43-year-old female with a family history of various gastrointestinal malignancies, together with breast, lung and parathyroid tumors. She has *MLH1* variant NM_000249.4:c.1836_1839del: p.(Val612del), which is a fs deletion, confirmed by expert panel to be LP in Clinvar.
- Patient #14 (Table 1) is a 9-year-old with a HGG and a family history of CRC, uterine cancer in the mother and thyroid tumors. She has a *PMS2* variant NM_000535.7: c.1269dup: p.(Gln424AlafsTer12). As endometrial cancer is an integrate tumor of LS, it is likely that the mother is a carrier of this variant.

Three missense (ms) variants were identified across three families, including two families with affected sibling pairs (five patients in total). These included a P *MSH6* variant, a LP *PMS2* variant, and a VUS in *MSH6*:

- Patient #11 (Table 1) is a 17-year-old girl who presented with a HGG, carries a pathogenic *MSH6* variant NM_000179.3:c.2314 C > T: p.(Arg772Trp), which is confirmed as P variant in Clinvar. Her parents have no history of cancer; however, the family is consanguineous. Notably, the maternal grandmother was diagnosed with uterine cancer at age 60, and the paternal grandfather developed colorectal cancer at age 40. This background highlights the need for genetic screening of both currently healthy parents.
- Patients #6 and #7 (Table 1) are sisters aging 13 and 15 years when presented with CRC, who are identified to carry an ms variant of *PMS2* gene NM_000535.7:c.903G > T: p.(Lys301Asn), classified as LP in Clinvar. The 15-year-old girl had a pilomatrixoma as well, and they had a family history of leukemia, uterine, pancreatic and skin malignancy. Similar to patient #11, the parents must have been yet undetected carriers of one of the *PMS2* variants. Although the children’s parents were not tested yet it is conceivable that both of them could be carrier of this *PMS2* missense variant.
- Patients #12 and #13 (Table 1) are siblings: a 22-year-old brother diagnosed with CRC and HGG, and his 13-year-old sister diagnosed with HGG. Their mother died from a brain tumor, and two maternal uncles were also diagnosed with brain tumors in their twenties. Based on this strong family history, the siblings were enrolled in the study despite negative IHC results for MMRD. Germline analysis came back positive only for a shared VUS in *MSH6* gene, NM_000179.3: c.1217G > A: p.(Cys406Tyr), which makes it worth reporting.
- Patient #2 (Table 1) is a 30-year-old female, who is diagnosed with CRC. er family history is highly suggestive of a hereditary CPS with several family members affected with gastrointestinal, uterine, sarcomas and other malignancies. No SNVs or indels were identified in her sample by sequencing. A large deletion of approximately 74 kb in *MSH2* was suggested by lcWGS (see sequencing artifacts section below), and MLPA analysis subsequently confirmed a heterozygous deletion involving exon 7 only (Supplementary Information (SI)). This deletion represents a known pathogenic copy number variant predicted to cause a frameshift and is associated with LS [11–13].

### Additional testing for patients without validated germline MMR variants

**Fig. 1:**
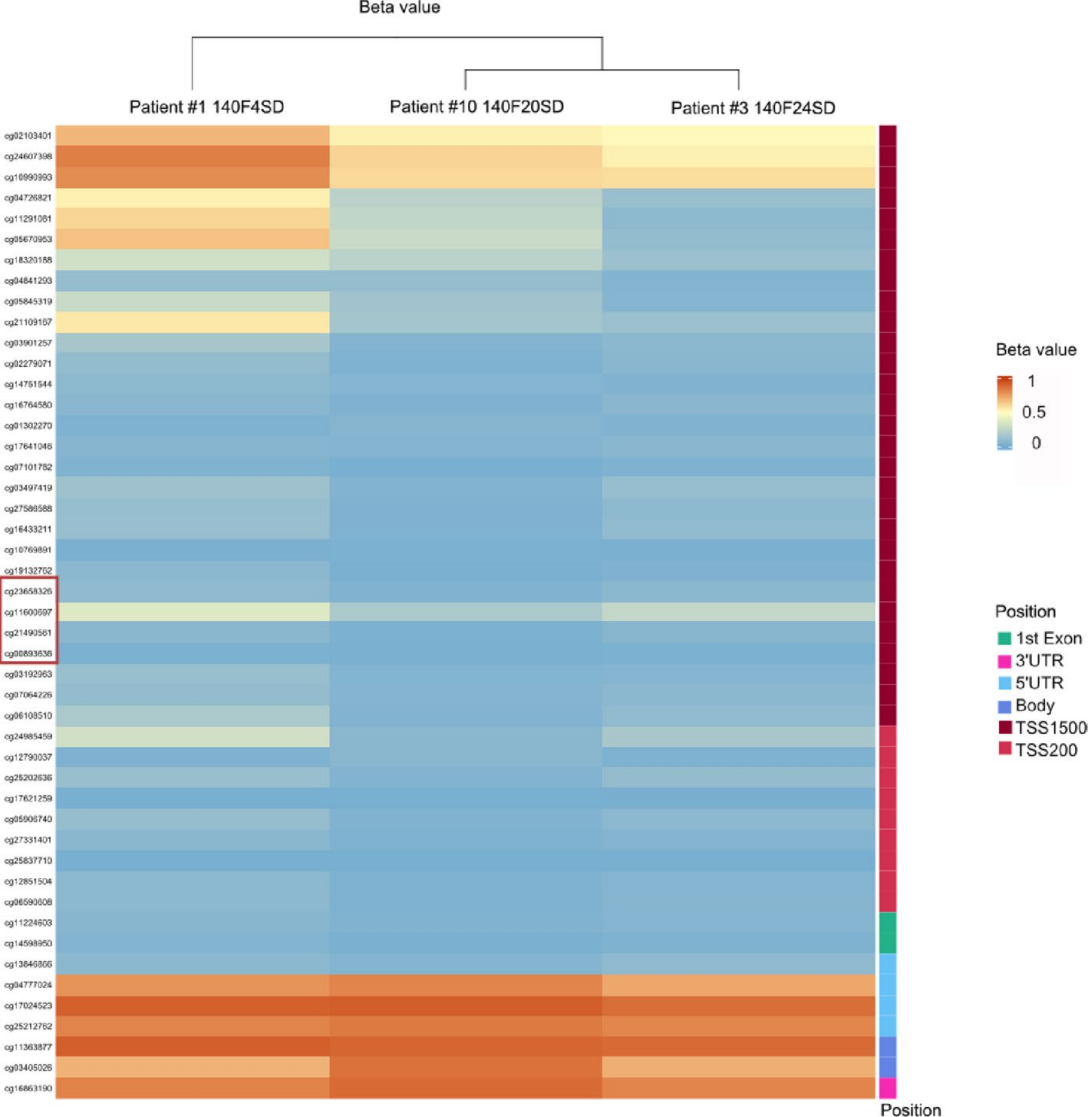
MLH1 promoter methylation analysis for patients #1, #3, and #10 showing absence of methylation at four relevant CpG sites (cg23658326, cg11600697, cg21490561, and cg00893636), indicated by the red box at he left side of the graph

For the two siblings (Patients #12 and #13) carrying only an *MSH6* variant of uncertain significance (NM_000179.3):c.1217G > A: p.(Cys406Tyr), as well as Patients #1, #3, and #10 (Table 1), in whom no pathogenic germline SNVs, indels, or CNVs affecting *MMR* genes were identified, germline DNA was further analyzed using a 213-gene CPS panel. No additional pathogenic or likely pathogenic (P/LP) variants were detected. For the three adult patients with CRC (Patients #1, #3, and #10), *MLH1* promoter methylation analysis was also performed and was negative in all cases (Fig. 1).

In addition, somatic WES data from all five patients were evaluated for SNVs, indels, CNVs affecting *MMR* genes, and *BRAF* V600E. A candidate somatic *MSH6* structural inversion involving exon 2 – intron 2 (chr2:48,018,253–48,018,370; GRCh37), supported by three sequencing reads, was identified in Patient #1. Patient #3 harbored a candidate somatic *MLH1* inversion involving intron 12–exon 13 (chr3:37,070,267–37,070,403; GRCh37), supported by three to four sequencing reads. No P/LP somatic *MMR* alterations or *BRAF* V600E variants were identified in Patients #10, #12, or #13.

Taken together, these findings support a diagnosis of suspected somatic MMR deficiency in Patients #1 and #3. In contrast, no alternative molecular explanation was identified for the two siblings carrying the homozygous *MSH6* p.Cys406Tyr variant, leaving open the possibility that this variant contributes to their phenotype, although functional validation is required. Similarly, no germline or somatic MMR alterations were identified in Patient #10, supporting classification as a sporadic CRC of unresolved molecular etiology.

### Apparent large deletions likely representing sequencing artifacts

**Fig. 2:**
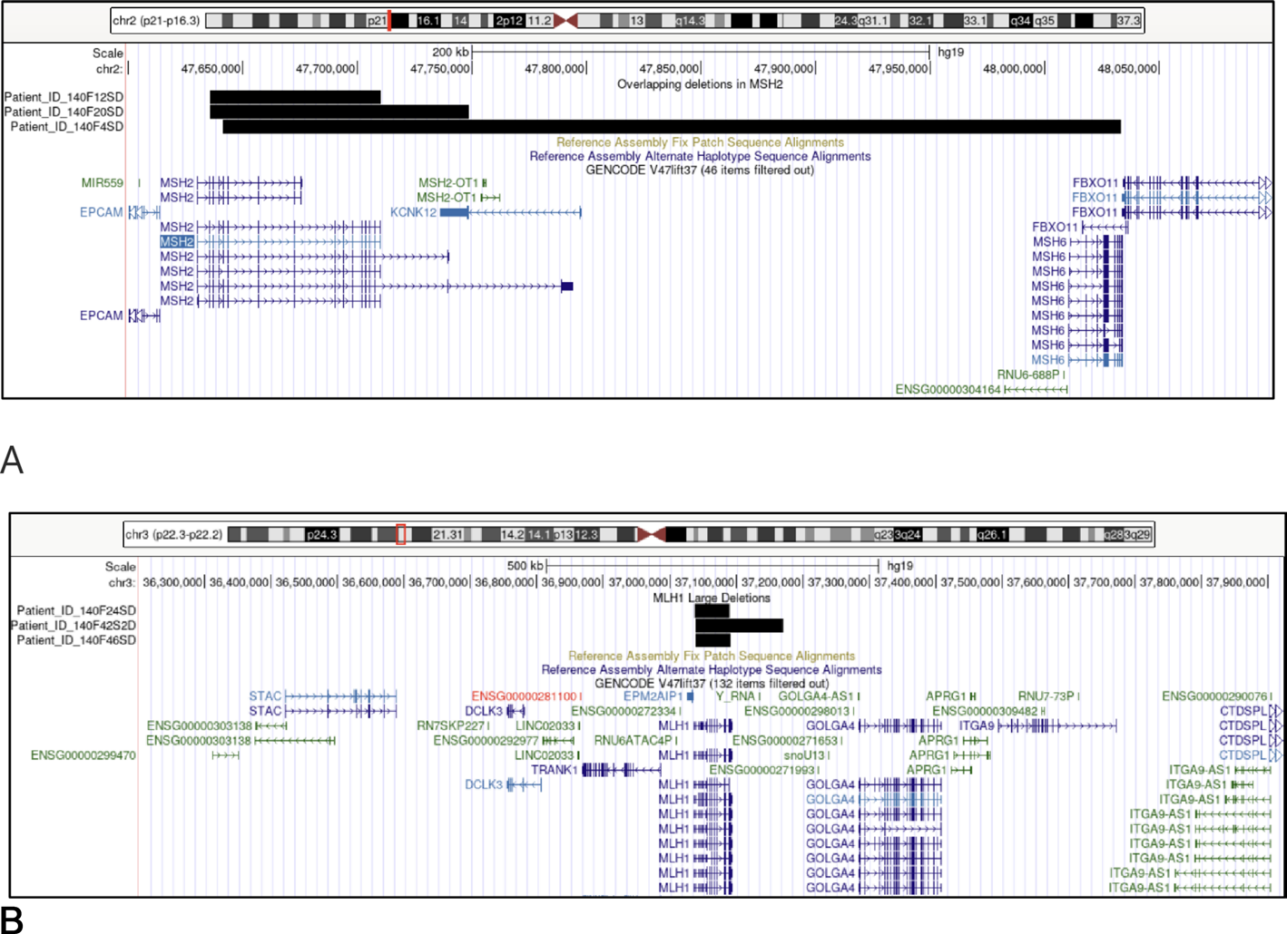
**A** Apparent overlapping deletions of MSH2 gene detected in three unrelated Jordanian patients as visualized on UCSC genomic browser, likely representing artifacts. **B** Apparent overlapping deletions of MLH1 gene detected in three unrelated Jordanian patients as visualized on UCSC genomic browser, likely representing artifacts

In addition to the identified sequence variants, exome-based CNV analysis using CNVkit suggested large deletions in six patients: three involving *MSH2* and three involving *MLH1* (supplementary Information); however, the large deletion events inferred from these plots were generally not supported by the lcWGS copy-number data. MLPA analysis confirmed only part of one deletion in a single patient (patient #2, Table 1), suggesting that most of these findings may represent sequencing artifacts rather than true constitutional deletions. Furthermore, the three cases with presumed *MSH2* deletion have overlapping segment of 74.6 kb (chr2:47,635,736–47,710,309, GRCh37) (Fig. 2-A), and the three patients with presumed *MLH1* deletion have an overlapping area spanning approximately 53 kb (chr3:37,037,285–37,090,106, GRCh37) (Fig. 2-B), originally calculated from WES- based BAF plots. The recurrence of highly overlapping genomic regions across unrelated patients further supports the interpretation of these events as technical artifacts. This conclusion is also consistent with the overall clinical and molecular characterization of the tumors, as discussed below on a case-by-case basis.

The following cases showed putative artifacts in *MSH2. BAF plots for all patients are available in SI*:

- Patient #1 (Table 1) exhibited a deletion spanning approximately 390 kb (chr2:47,641,377–48,033,551, GRCh37), resulting in loss of *MSH2* from intron 4 onward as well as deletion of the first eight exons of *MSH6*. As explained in previous paragraph, no additional molecular events were identified: specifically, no SNVs or indels in the MMR genes, no pathogenic variants in other CPS genes, and no *MLH1* promoter hypermethylation. The absence of strong clinical suspicion and patient’s enrolment based solely on young age (39 years), the IHC staining pattern could be attributable to a somatic event.
- Patient #2 (Table 1) showed a 74 kb deletion (chr2:47,635,736–47,710,309, GRCh37), which encompasses most of the gene from intron 2 to parts of the 3’UTR of *MSH2* and overlaps with the first deletion. MLPA analysis carried out with two different probe kits only confirmed a large deletion of exon 7 and of 51nt of DNA downstream of exon 7 exclusively, as explained above.
- Patient #10 (Table 1) presented with a 112 kb deletion (chr2:47,635,736–47,748,646, GRCh37) encompassing intron 2 of *MSH2* and extending into the *KCNK12* gene, overlapping part of the deleted region observed in Patients #1 and #2. As explained in previous paragraph, and similar to patient #1, no additional molecular germline events were identified. Apart from his age at diagnosis (38 years), this patient did not exhibit strong clinical suspicion for a hereditary CPS. His tumor mutational burden was low (TMB 2.1), and MSI status was also low (0.13%). The loss of MMR protein expression on IHC may therefore reflect a somatic rather than germline event.

The following cases showed putative artifacts in *MLH1. BAF plots for all patients are available in SI*:

- Patient #3 (Table 1) exhibited a deletion spanning approximately 53 kb (chr3:37,037,285–37,090,106, GRCh37), affecting exons 2–17 (parts of intron 1 to parts of intron 17). As explained in previous paragraph, and similar to patients #1 and 10, no additional molecular germline events were identified. Given the absence of strong clinical suspicion and his enrolment based solely on young age (44 years), the IHC staining pattern could be attributable to a somatic event.
- Patient #13 (Table 1) showed an approximately 132 kb (chr3:37,038,204–37,170,640, GRCh37), encompassing most parts of intron 2 far into the 3’ untranslated region (UTR) and the noncoding *UBE2F* pseudogene 1 (*UBE2FP1*). However, the patient’s sibling (patient #12, Table 1) did not show the same deletion. Both siblings had normal IHC despite the suggestive clinical picture of CMMRD. Indeed, both siblings showed a VUS in *MSH6* gene (NM_000179.3): c.1217G > A: p.(Cys406Tyr), which might be causative of their clinical picture.
- Patient #11 (Table 1) showed an approximately 54 kb in size (chr3: 37,038,204–37,093,204, GRCh37), affecting most parts of intron 2 to the 3’ UTR. This variant cannot explain the IHC loss of *MSH2*, *MSH6*. In addition, this patient had a P variant of *MSH6* gene (NM_000179.3): c.2314 C > T: p.(Arg772Trp), which clearly explains the patient’s clinical picture including her diagnosis with HGG at the age of 17 years among other features.

### Additional molecular findings

TMB varied significantly among patients, ranging from 2.1 to 352 variant/Mb, with a median of 23 variant/Mb and a mode of 19 variant/Mb. Notably, four tumors exhibited a TMB of less than 10 variant/Mb, while one had a TMB exceeding 100 variant/Mb.

MSI on the basis of NGS data of WES was low in eight of 14 patients, including six of the proved 11 MMRD tumors, with scores ranging from 0.13% to 26% (mean: 10.6%). This limitation might be related to FFPE material as explained in the discussion below.

Mutational signature analysis identified the ubiquitous clock-like signatures SBS1 and SBS5 in most tumors, with SBS5 present in all cases (Table 1 and Supplementary Information). In addition, tumors with confirmed or suspected MMR deficiency demonstrated characteristic MMRD-associated signatures, including SBS6, SBS15, SBS20, SBS26, and SBS44 [14]. The two patients with suspected somatic MMRD (Patients #1 and #3) both exhibited SBS6, consistent with defective mismatch repair. Similarly, most patients with confirmed or likely CMMRD showed predominant SBS15, with additional contributions from SBS20, SBS26, or SBS44 in individual cases. In contrast, the single MMR-proficient CRC (Patient #10) demonstrated only the clock-like SBS1 and SBS5 signatures and lacked MMRD-associated mutational signatures.

Interestingly, the single ultra hypermutated tumor (patient #13) had mutational signature suggestive of combined MMRD and POLE mutation (SBS15, SBS14, SBS10a and SBS10b, Supplementary Information). Further analysis of somatic WES data has identified *POLE*(NM_006231.4): c.1231G > T: p.(Val411Leu).

## Discussion

Nine of the fourteen enrolled patients harbored a pathogenic or likely pathogenic germline variant in an MMR gene. In addition, two affected siblings carried a homozygous *MSH6* variant of uncertain significance p.(Cys406Tyr), for which no alternative molecular explanation was identified. Two patients (Patients #1 and #3) lacked validated germline *MMR* alterations but demonstrated candidate somatic structural rearrangements involving *MSH6* and *MLH1*, respectively, supporting a diagnosis of suspected somatic *MMR* deficiency. In contrast, Patient #10 showed no germline or somatic *MMR* alterations, no *MLH1* promoter hypermethylation, and no *BRAF* V600E mutation, and was therefore classified as a sporadic CRC of unresolved molecular etiology.

Indeed, a substantial proportion of patients fulfilling the clinical criteria for LS or CMMRD remain without an identifiable pathogenic germline MMR variant despite comprehensive molecular testing. Previous studies of 28 and 38 patients with clinically suspected CMMRD reported that 25% and 26% of cases, respectively, lacked an identifiable pathogenic or likely pathogenic germline MMR variant. Our findings are consistent with these observations and further illustrate the molecular heterogeneity of MMR-deficient tumors, emphasizing the importance of integrating clinical features with germline and somatic genomic analyses [8]. Similarly, it is estimated that only 53–70% of suspected LS cases show germline variants, with the higher end of the range relating to cases with IHC loss of *MSH2*/*MSH6* [15]. Other cancer predisposition genes explained some cases [15]. In our cases series, all the three patients for whom no variants were identified, have weak clinical suspicion and were enrolled because of their age, and suggestive IHC results. Nevertheless, we examined them for other CPS and negative results were obtained. In addition, none of those cases showed evidence of *MLH1* promoter hypermethylation.

The two siblings (patients #12 and #13) exhibit strong clinical suspicion for CMMRD despite negative IHC stains of *MMR* genes, with no SNV, indel or CNV of MMR genes identified. The fact that both of them carry the same VUS variant of *MSH6* gene (NM_000179.3):c.1217G > A: p.(Cys406Tyr) makes it worth reporting. Functional genomic testing for this variant can be helpful to confirm the diagnosis [16].

Apparent large deletions likely representing sequencing artifacts were identified in 6 patients, 3 showing overlapping deletions of *MLH1* and 3 showing overlapping deletions in *MSH2*. At first, the deletions looked similar to each other which raised our suspicion for artifacts (BAF plots, Supplementary Information). Moreover, two patients already carry other germline MMR events that could explain their situation (patients #11 and #13). Accordingly, MLPA [17], which is a sensitive technique for detecting large CNVs through quantitative analysis of specific regions of the *MMR* genes was conducted. Only one patient of the six proved to have part of the identified sequencing deletion of *MSH2* confirmed by MLPA, leaving three cases (patient #1, #3 and #10, Table 1) suspected to be somatic MMRD given the absence of other germline events and the weak clinical suspicion.

TMB in our cohort exhibited a wide range, with four tumors having normal TMB unexpectedly, with only one of which suspected to be a somatic case of CRC (patient #10). The majority displayed hypermutation (> 10 variants/Mb) and one was classified as ultra-hypermutated (> 100 variants/Mb). Hypermutation and ultra-hypermutation have been strongly linked to MMRD, which are characteristic of LS and CMMRD, with the latter being more likely to demonstrate extreme mutational loads [8, 18]. Unexpectedly, six of the proved MMRD tumors of 11 patients exhibited low MSI. This might be related to the quality of DNA extracted from old FFPE blocks dating back to 1991 for the oldest block (patient #5, Table 1). According to Qingli Guo et al. “Application of MSIsensor to an unrepaired CRC sample could lead to miscalling of MSI status for the tumour” [19]. Additionally, tumor purity (percentage of tumor cells per slide) is a prerequisite for successful MSI analysis [20]. Utilizing the more sensitive Low-Pass Genomic Instability Characterisation Assay (LOGIC) would be helpful in such scenario [21].

An interesting observation from the mutational signature analysis was the marked difference between the two siblings carrying the homozygous *MSH6* p.Cys406Tyr variant. Although both tumors demonstrated MMRD-associated mutational signatures, Patient #13 was the only ultrahypermutated case in the cohort (TMB 352 variants/Mb) and exhibited a mutational signature profile consistent with combined mismatch repair and DNA polymerase proofreading deficiency, including SBS14, SBS10a, and SBS10b. In contrast, the sibling’s tumor showed a substantially lower TMB (22.8 variants/Mb) and lacked these polymerase-associated signatures. Further review of the somatic WES data identified the canonical pathogenic *POLE* exonuclease-domain hotspot mutation *POLE* (NM_006231.4):c.1231G > T: p.(Val411Leu) in Patient #13. This observation is consistent with the proposed stepwise model of tumor evolution in replication repair-deficient cancers, in which a primary MMR defect is followed by acquisition of a secondary somatic *POLE* proofreading mutation, resulting in combined MMR and polymerase proofreading deficiency and the development of an ultrahypermutated phenotype [22]. These findings highlight the biological heterogeneity that can occur even between affected siblings carrying the same underlying germline MMR defect.

Finally, this study highlights the importance of surveillance in affected Jordanian families. Notably, the presence of a germline MMR variant in affected children may serve as an indicator of cancer risk in their often asymptomatic parents. This is illustrated by patients #6, #7 and #11, who developed tumors while parents were still clinically unaffected. In addition, the study underscores the limitations of using FFPE material for molecular characterization of MMRD tumors and highlights the need to confirm sequencing-based CNVs by MLPA analysis. Another limitation is the unavailability of parental DNA samples, which precluded discrimination between biallelic and monoallelic inheritance.

## Supplementary Information

Supplementary Material 1. BAF_Plots.PDF includes: Figure 1: B allele frequency (BAF) plots of patients #1, #10 and #2 with apparent large
deletions of MSH2 gene, that look relatively similar to each other, likely representing sequencing artifacts. Figure 2: B allele frequency (BAF) plots of patients #3, #13 and #11 with apparent large deletions that look relatively similar to each other, likely representing sequencing artifacts.

Supplementary Material 2. CPS_panel_genes. docs includes Table 1: List of genes analyzed using the cancer predisposition sequencing (CPS) panel.

Supplementary Material 3. MLPA_MSH2del7.pdf includes: MLPA Report.

Supplementary Material 4. SI_Signature Contributions.csv, includes: Table of percentages of COSMIC signature contributions for each tumor.

Supplementary Material 5. SI_Mutational Signatures. PDF includes: Figure: Mutational Signatures of Tumors Included in the Study.

Supplementary Material 6. SI_Pedigrees.PDF includes: Figure: Pedigrees of Patients included in the study.

## Data Availability

DNA sequencing data were deposited into the German Human Genome-Phenome Archive GHGA repository under accession number GHGAS19356866116009 and are available at the following URL: [https://data.ghga.de/study/GHGAS19356866116009](https:/data.ghga.de/study/GHGAS19356866116009)Access is subject to controlled access procedures in accordance with GHGA data governance policies.

## Abbreviations

LS: Lynch syndrome
CPSs: Cancer predisposition syndromes
P: Pathogenic
LP: Likely pathogenic
MMR: Mismatch repair
CMMRD: Constitutional mismatch repair deficiency
MMRD: Mismatch repair deficiency
CRC: Colorectal carcinoma
IHC: Immunohistochemistry
HGG: High grade glioma
MSI: Microsatellite instability
TMB: Tumor mutational burden
KHCC: King Hussein Cancer Center
DKFZ: German Cancer Research Center
IRB: Institutional Review Board
FFPE: Formalin-Fixed Paraffin-Embedded
lcWGS: Low coverage whole genome sequencing
WES: Whole exome sequencing
UBE2FP1: UBE2F pseudogene 1
CNV: Copy number variant
BAF: B allele frequency
MLPA: Multiplex ligation-dependent probe amplification
QC: Quality control
SNV: Single nucleotide variant
UTR: Untranslated region
MSI-H: Microsatellite instability- high
fs: Frameshift
ms: Missense
SI: Supplementary Information
NGS: Next generation sequencing
