# Supplementary material for: Molecular characterization of mismatch repair deficient tumors in young Jordanian patients
Source: Discov Oncol. 2026 Aug 17;17:1198. doi: 10.1007/s12672-026-05717-3 (PMC13481598; doi:10.1007/s12672-026-05717-3)
Supplement: Supplementary file 1 [file 12672_2026_5717_MOESM1_ESM.pdf]

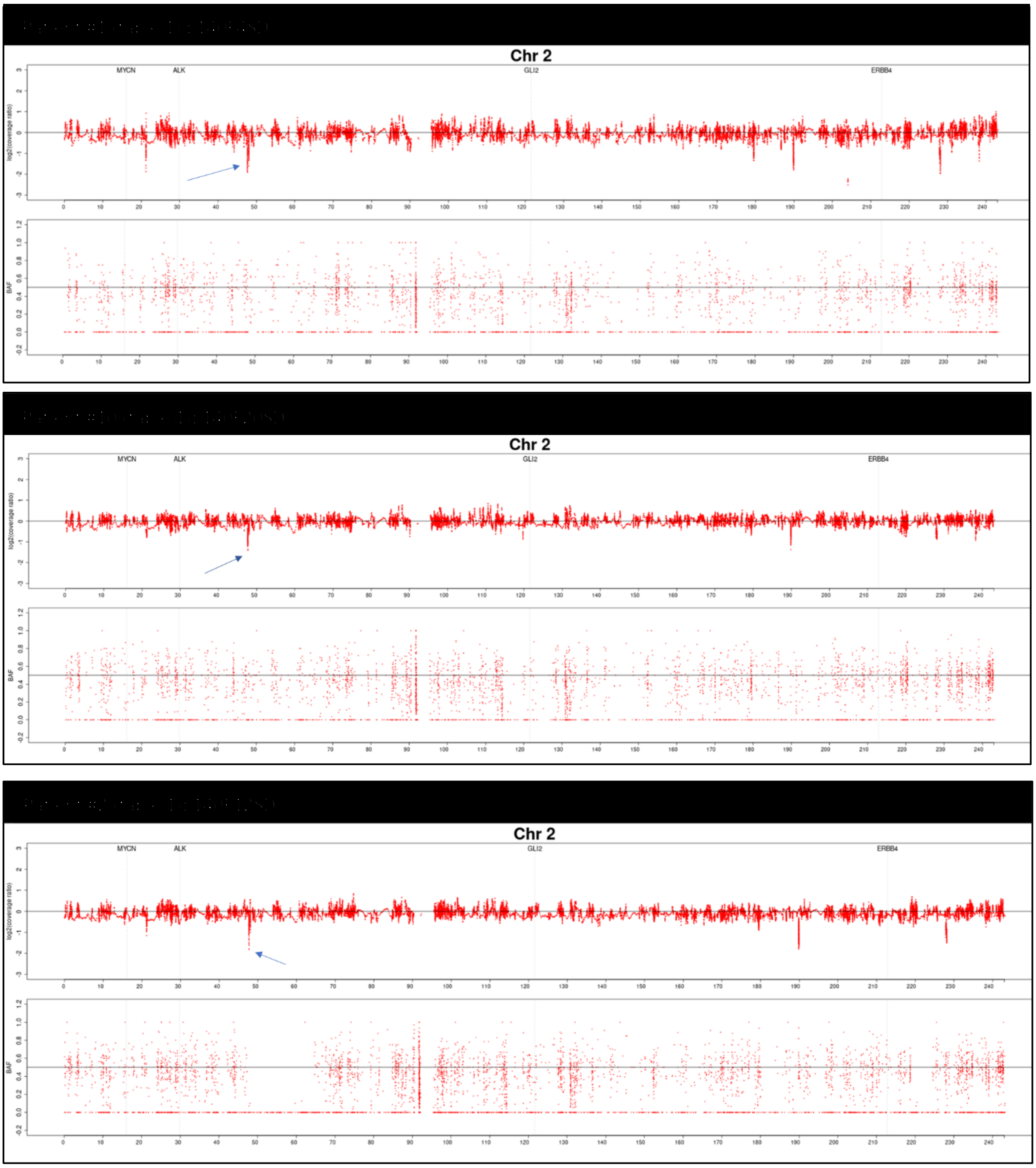

Figure 1: B allele frequency (BAF) plots of patients #1, #10 and #2 with apparent large deletions of MSH2 gene, that look relatively similar to each other, likely representing sequencing artifacts.

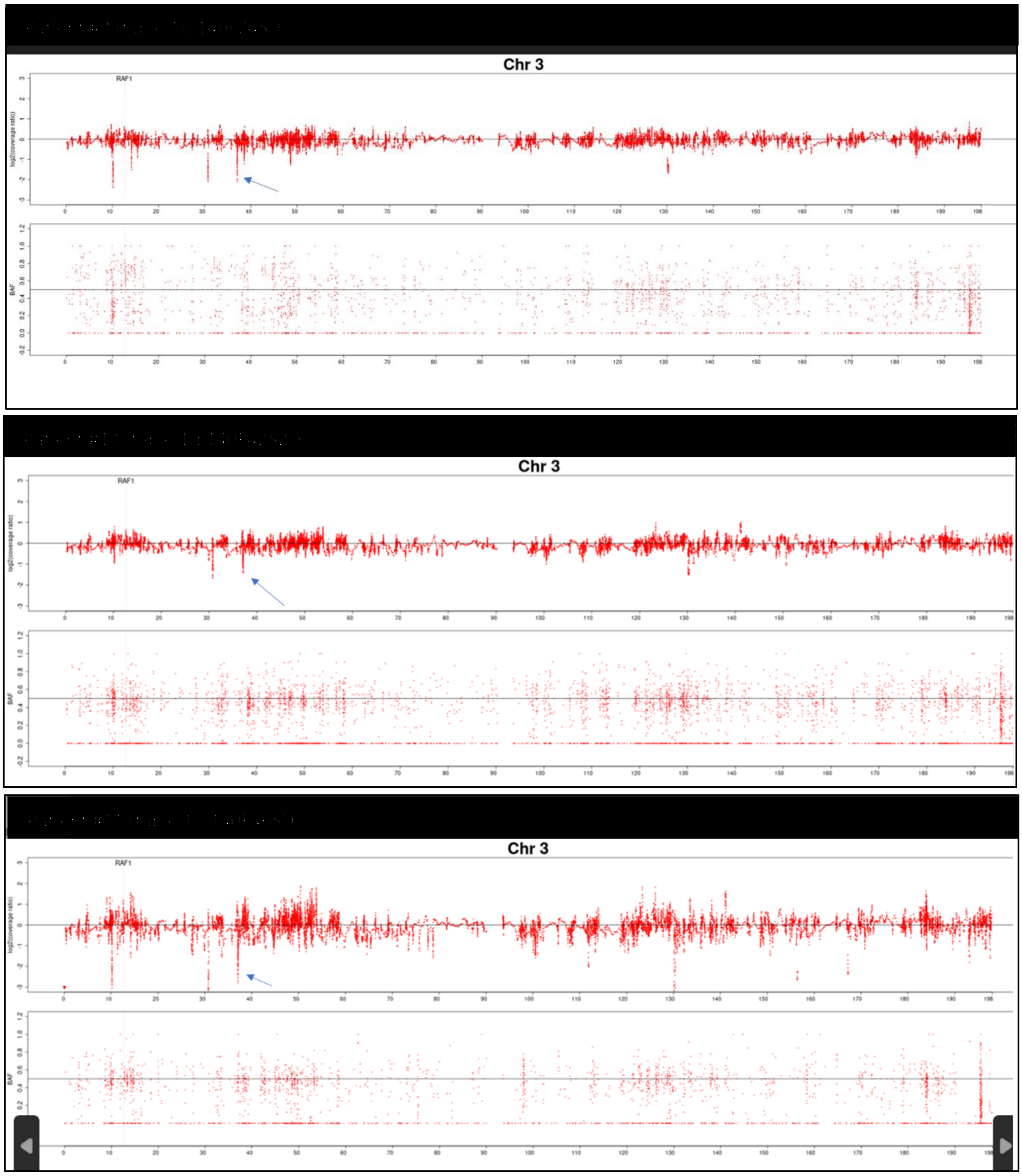

Figure 2: B allele frequency (BAF) plots of patients #3, #13 and #11 with apparent large deletions that look relatively similar to each other, likely representing sequencing artifacts.
