# Supplementary material for: Molecular characterization of mismatch repair deficient tumors in young Jordanian patients
Source: Discov Oncol. 2026 Aug 17;17:1198. doi: 10.1007/s12672-026-05717-3 (PMC13481598; doi:10.1007/s12672-026-05717-3)
Supplement: Supplementary file 2 [file 12672_2026_5717_MOESM3_ESM.pdf]

Institut für Humangenetik
Molekulargenetische Diagnostik
Im Neuenheimer Feld 366
D-69120 Heidelberg

JSI medical systems

MLPA REPORT

**Order-No :**	252170022	**DNA-No :**	140F12SDP	**Project :**	
**Patient :**	P-140F12SDP	**Patient-ID :**	140F12SDP	**Family-ID :**	
**MV :**	09/04/2025 Sutter, Christian (cs)				

Results:

**P248-B1**	**(140F12SDP_P248-B1_F_P)2_MLPA_20250826_IS_ABI**			**08/26/2025**	**All vs all**	
	deletion	02	p21	MSH2	E07	160
**P248-B1**	**(140F12SDP_P248-B1_F_P)1_MLPA_20250826_IS_ABI**			**08/26/2025**	**All vs all**	
	deletion	02	p21	MSH2	E07	160

**Resultfile:**	(140F12SDP_P248-B1_F_P)2_MLPA_202	08/26/2025 14:35:00	P248-B1		
**Control:**	(20711-1-1_P248-B1_F_C)_MLPA_202508	08/26/2025 14:35:00	P248-B1	compl. TV	tm [09/04/2025 12:54:21]
**Control:**	(20814-1-1_P248-B1_F_C)_MLPA_202508	08/26/2025 14:35:00	P248-B1	compl. TV	tm [09/04/2025 12:53:55]
**Control:**	(21550-1-1_P248-B1_F_C)_MLPA_202508	08/26/2025 14:35:00	P248-B1	compl. TV	tm [09/04/2025 12:53:34]

Analysis Mode: All vs all

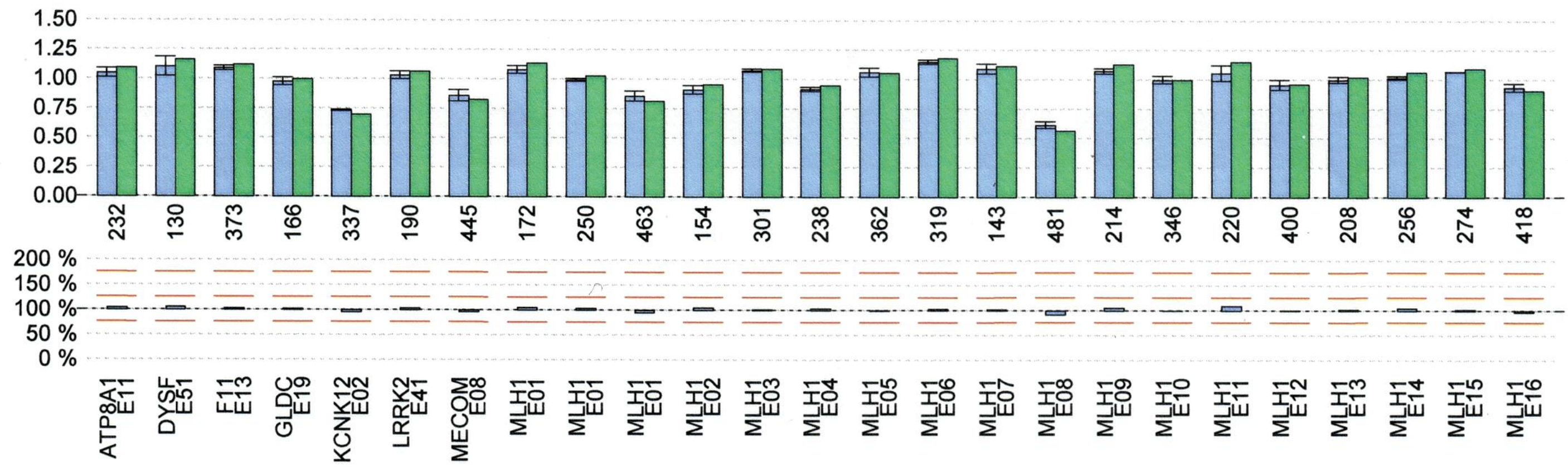

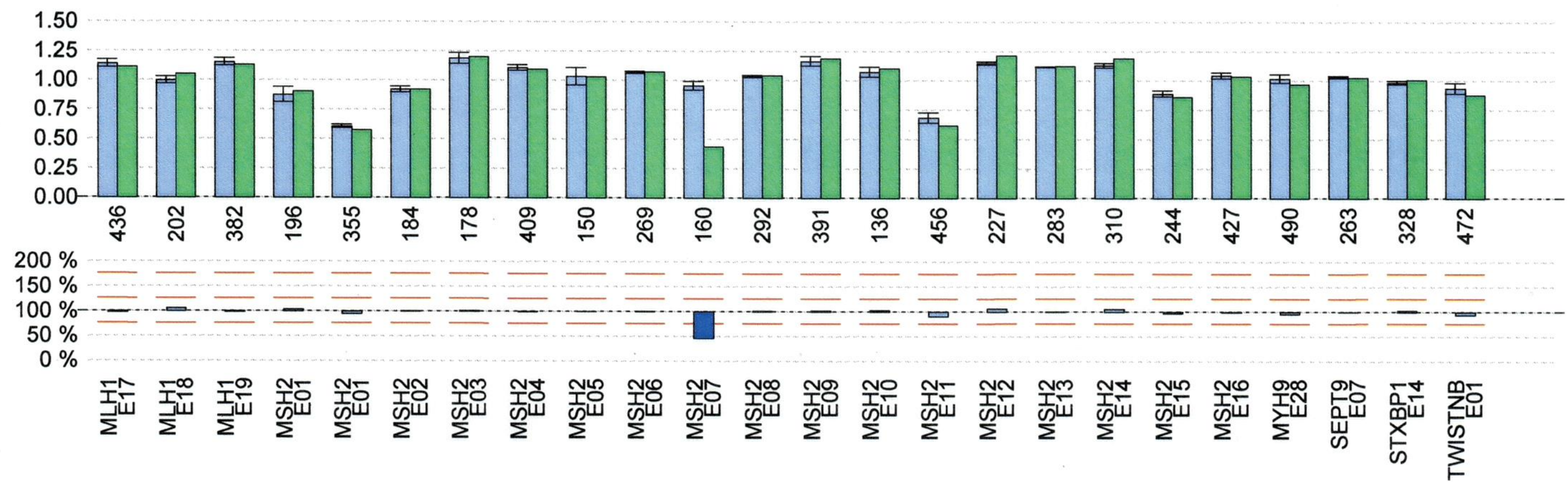

**Resultfile:**	(140F12SDP_P248-B1_F_P)1_MLPA_202	08/26/2025 14:35:00	P248-B1		
**Control:**	(20711-1-1_P248-B1_F_C)_MLPA_202508	08/26/2025 14:35:00	P248-B1	compl. TV	tm [09/04/2025 12:54:21]
**Control:**	(20814-1-1_P248-B1_F_C)_MLPA_20250È	08/26/2025 14:35:00	P248-B1	compl. TV	tm [09/04/2025 12:53:55]
**Control:**	(21550-1-1_P248-B1_F_C)_MLPA_20250È	08/26/2025 14:35:00	P248-B1	compl. TV	tm [09/04/2025 12:53:34]

Analysis Mode: All vs all

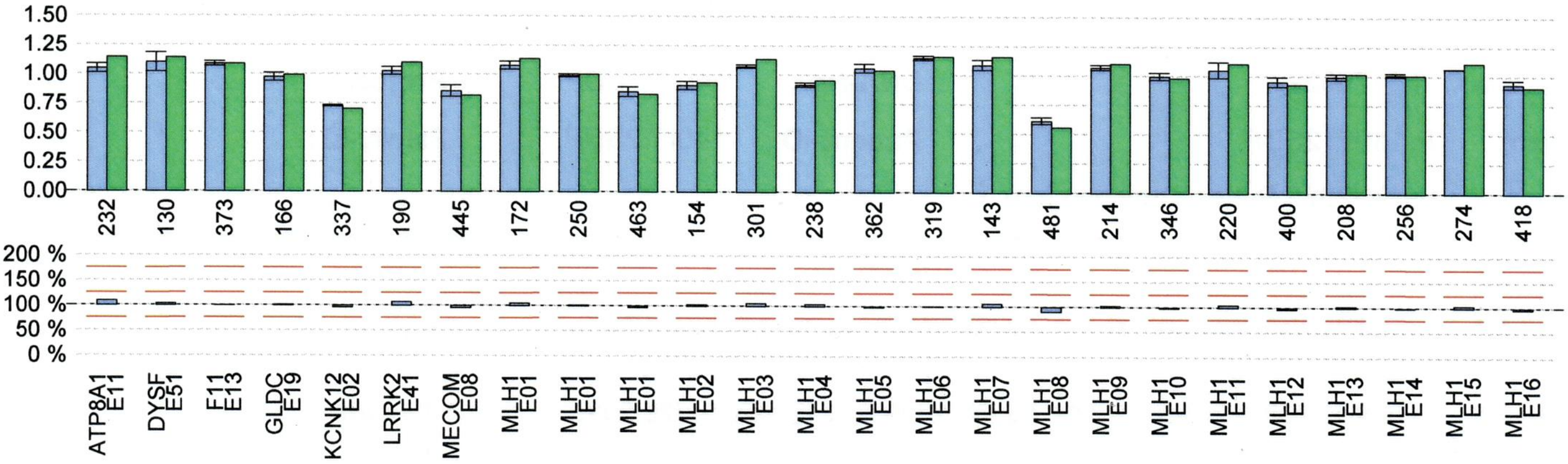

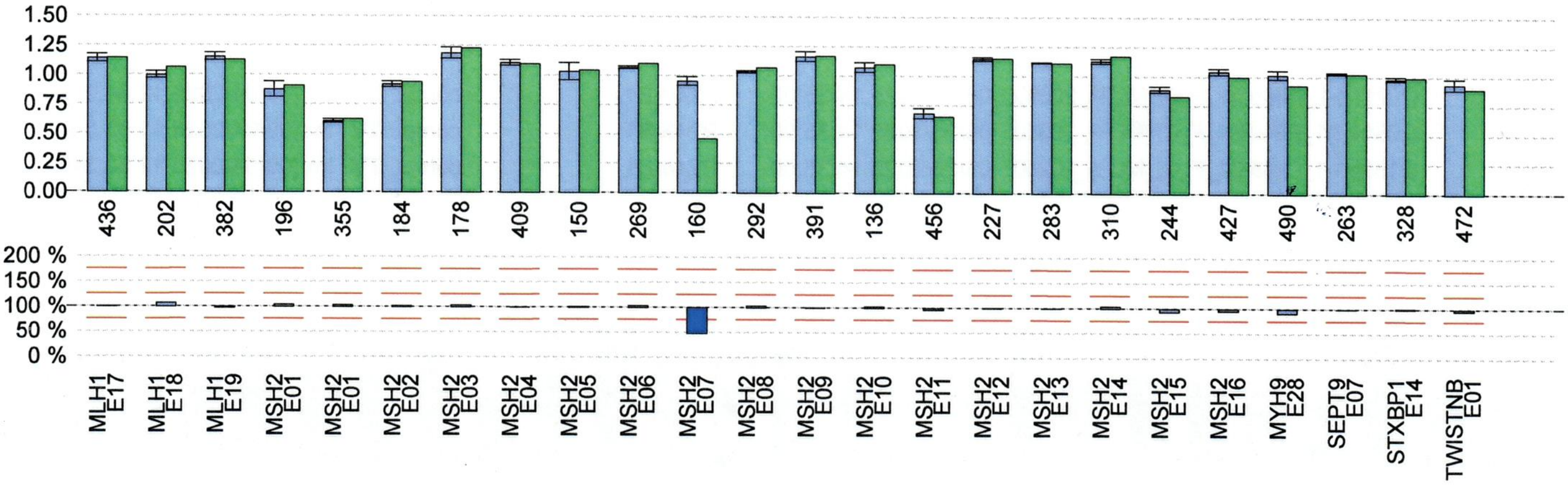

Comment:

Institut für Humangenetik
Molekulargenetische Diagnostik
Im Neuenheimer Feld 366
D-69120 Heidelberg

MLPA REPORT

Order-No : 252170022 **DNA-No :** 140F12SDP **Project :**

Patient : P-140F12SDP **Patient-ID :** 140F12SDP **Family-ID :**

MV : 08/19/2025 Sutter, Christian (cs)

Results:

**P003-D1**	**(140F12SDP_P003-D1_F_P)1_MLPA_20250819_IS_1_0**		**08/19/2025**	**All vs all**		
	deletion	02	p21	MSH2	E07	249

**Resultfile:**	(140F12SDP_P003-D1_F_P)1_MLPA_202	08/19/2025 12:57:00	P003-D1		
**Control:**	(20814-1-1_P003-D1_F_C)_MLPA_20250E	08/19/2025 12:57:00	P003-D1	compl. TV	is [08/19/2025 14:57:54]
**Control:**	(21550-1-1_P003-D1_F_C)_MLPA_20250E	08/19/2025 12:57:00	P003-D1	compl. TV	is [08/19/2025 14:59:30]
**Control:**	(20711-1-1_P003-D1_F_C)_MLPA_20250E	08/19/2025 12:57:00	P003-D1	compl. TV	is [08/19/2025 14:56:22]

Analysis Mode: All vs all

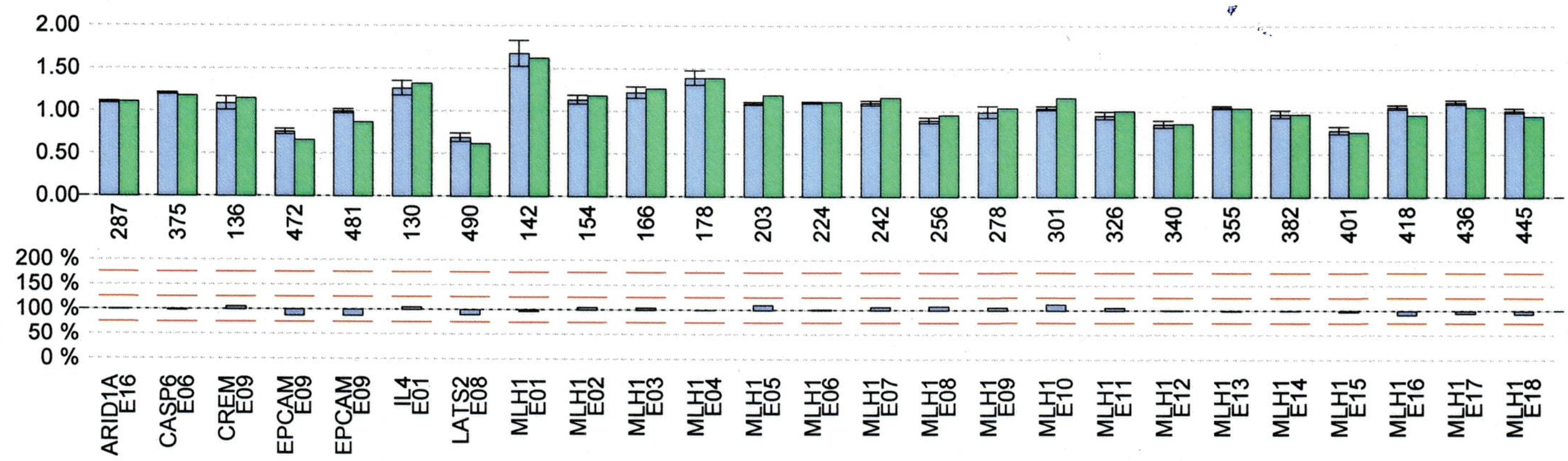

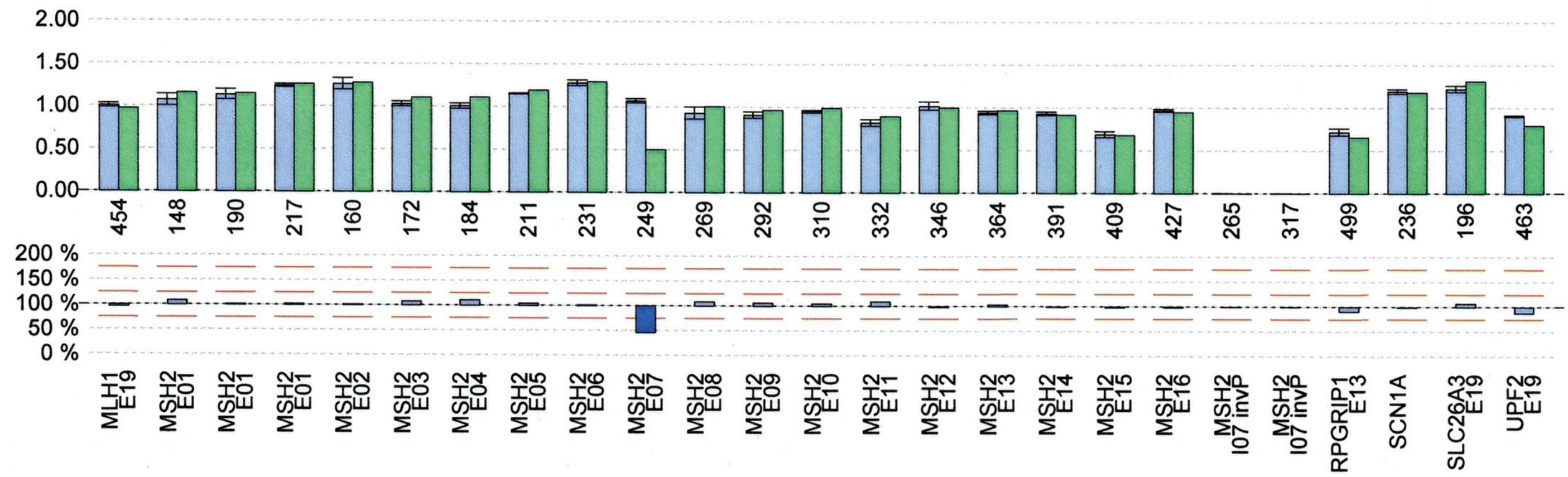

Comment:

Software Version: SEQUENCE Pilot 5.4.1

Print Date 19.8.2025
