# Supplementary material for: Molecular characterization of mismatch repair deficient tumors in young Jordanian patients
Source: Discov Oncol. 2026 Aug 17;17:1198. doi: 10.1007/s12672-026-05717-3 (PMC13481598; doi:10.1007/s12672-026-05717-3)
Supplement: Supplementary file 3 [file 12672_2026_5717_MOESM6_ESM.pdf]

Pedigrees of Patients included in the study

Patient #1 (140F4SD)

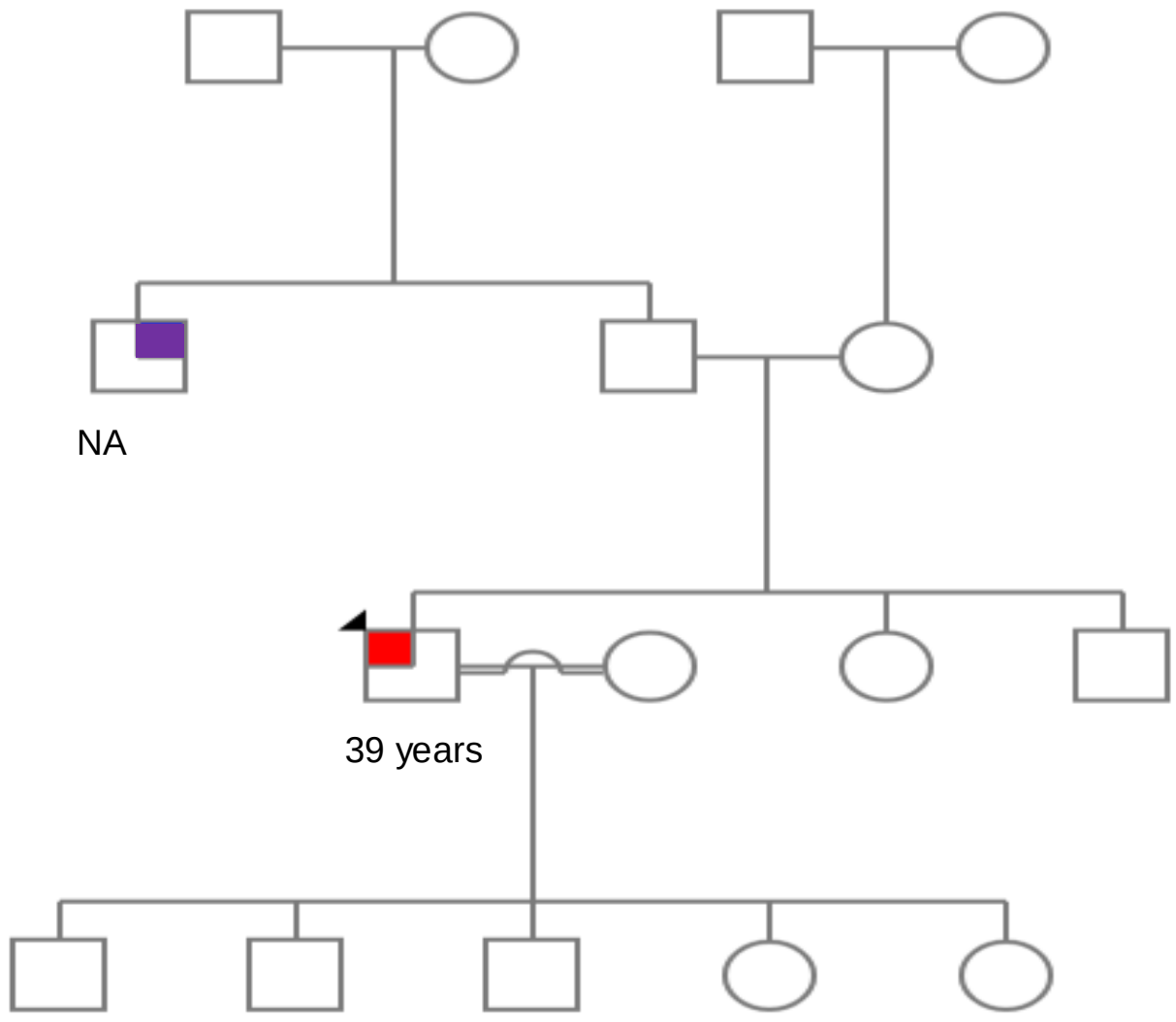

Patient #2 (140F12SD)

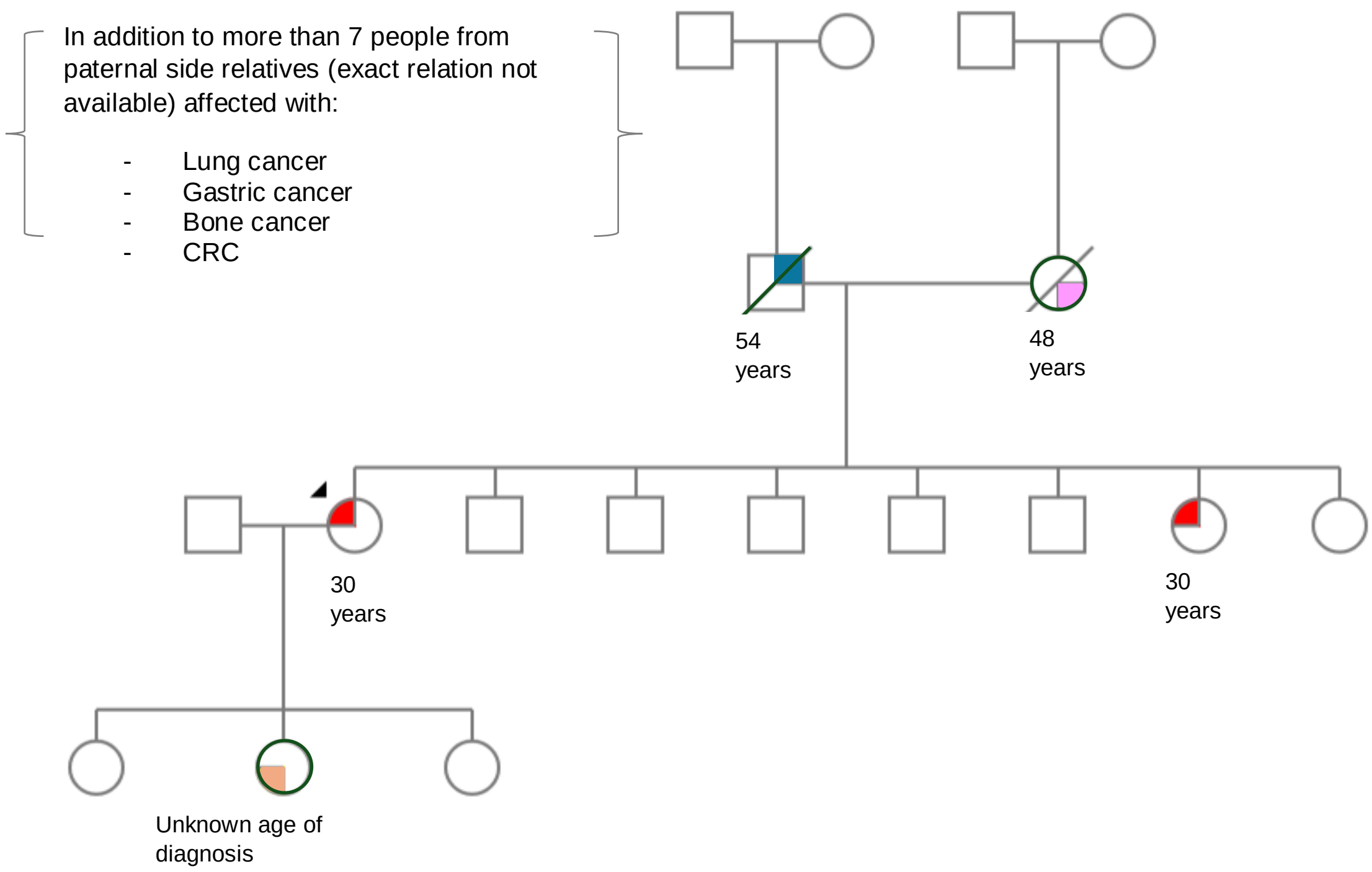

Patient #3 (140F24SD)

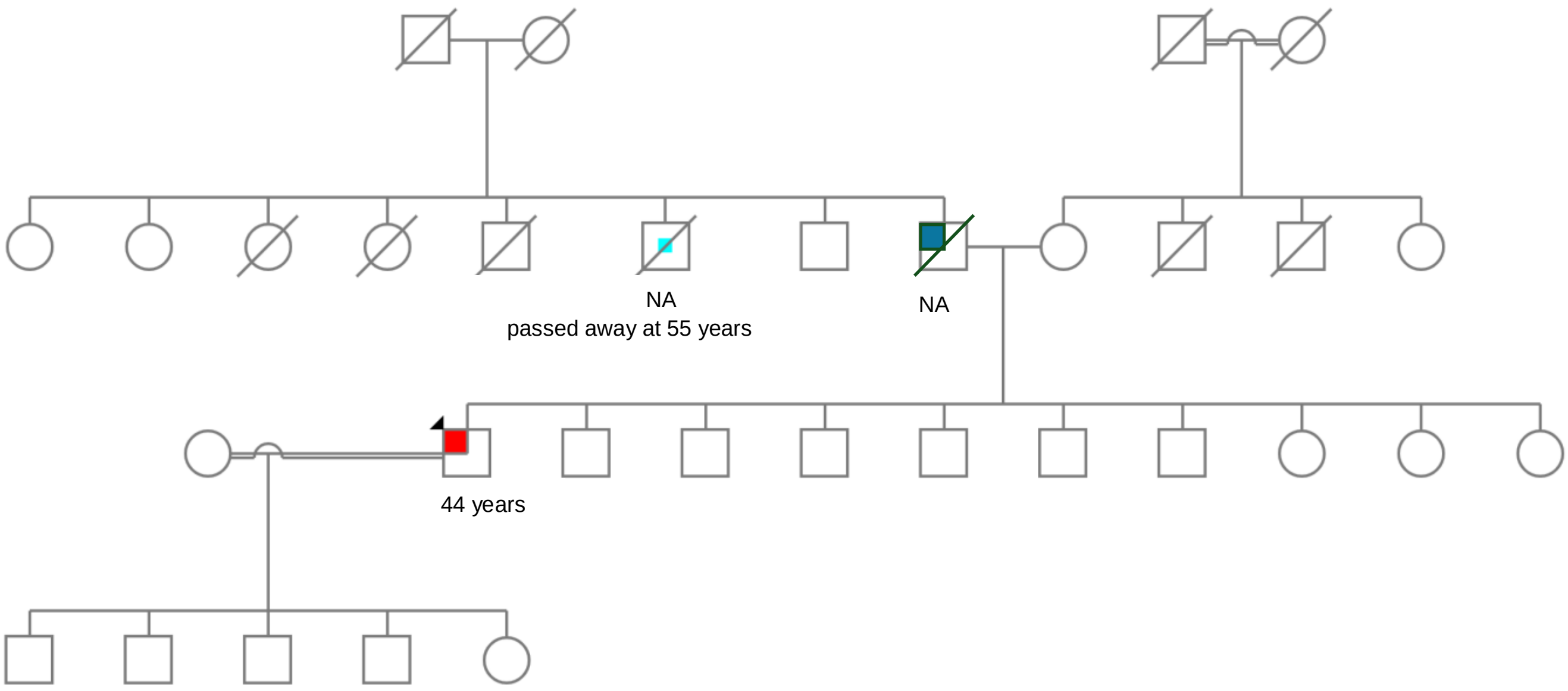

Patient #4 (140F2SD)

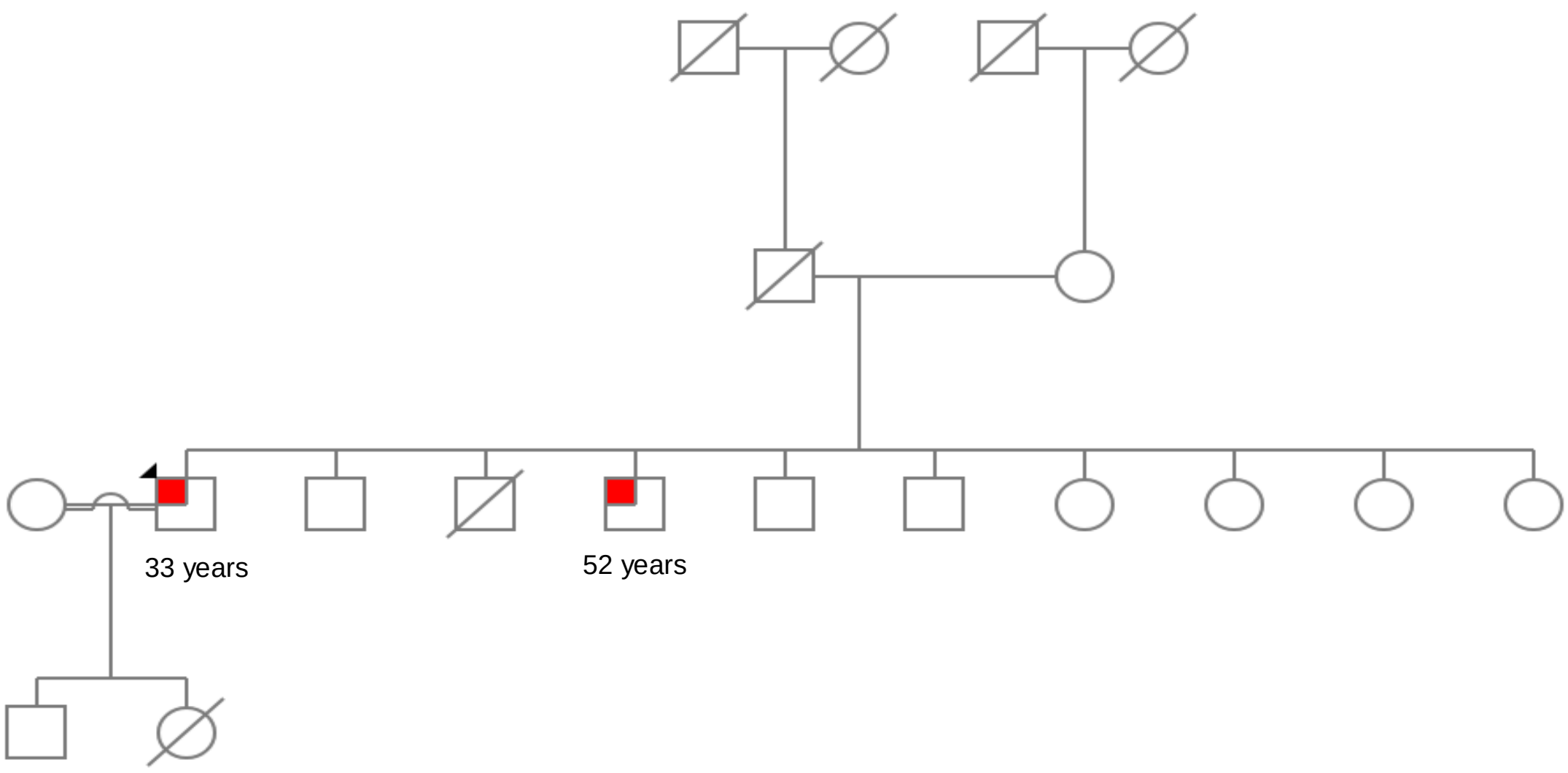

Patient #5 (140F30SD)

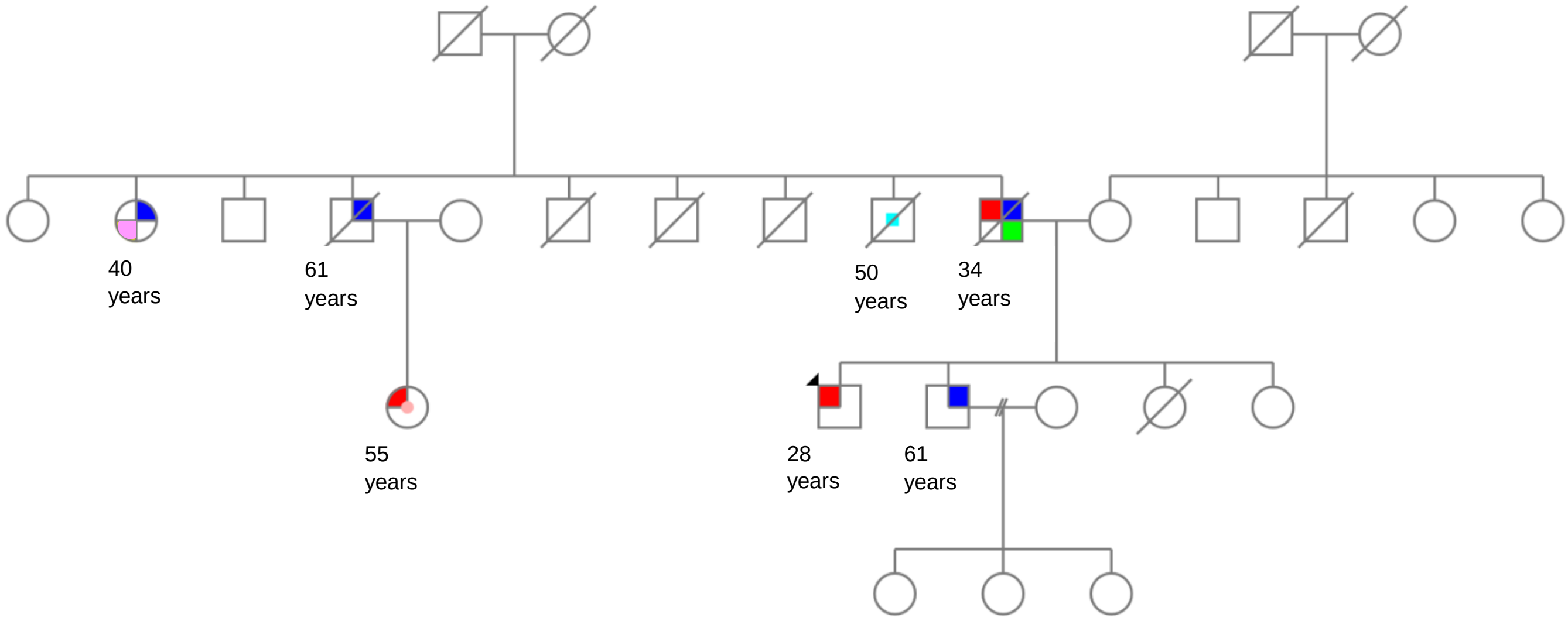

Patient #6&7 (140F29S1D & 140F29S2D)

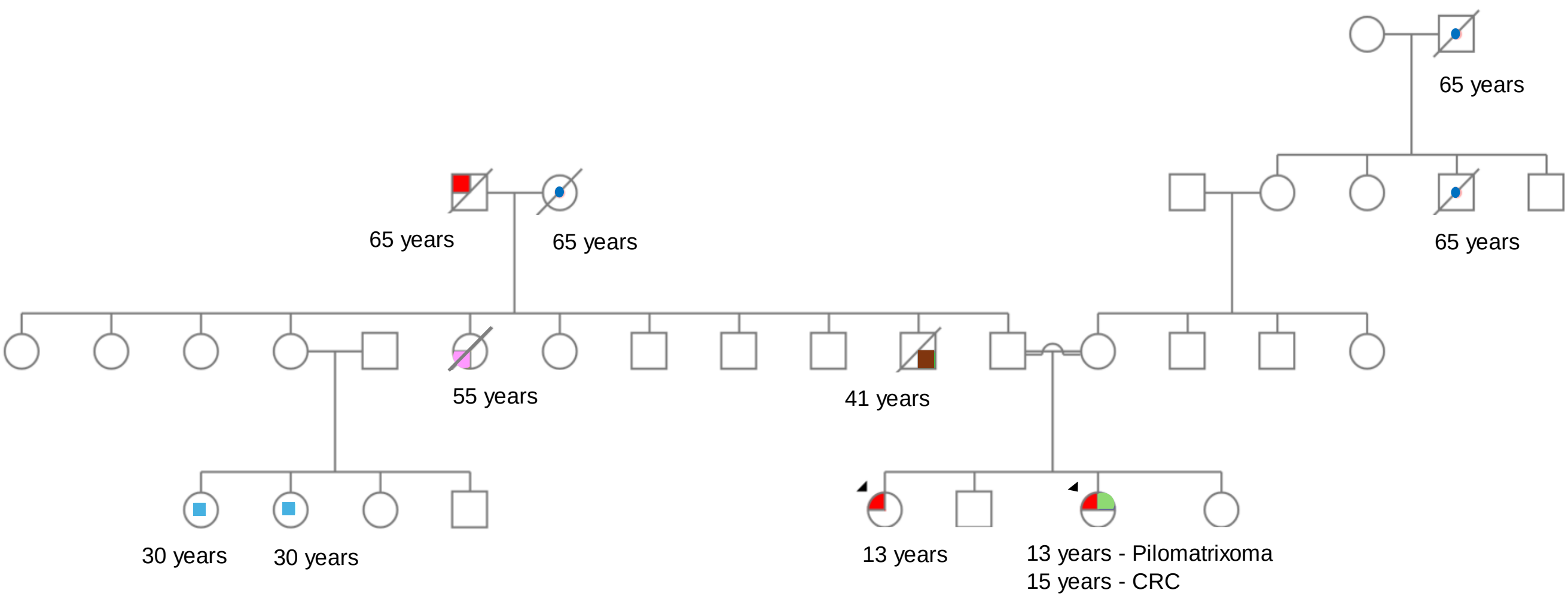

Patient #8 (140F19SD1)

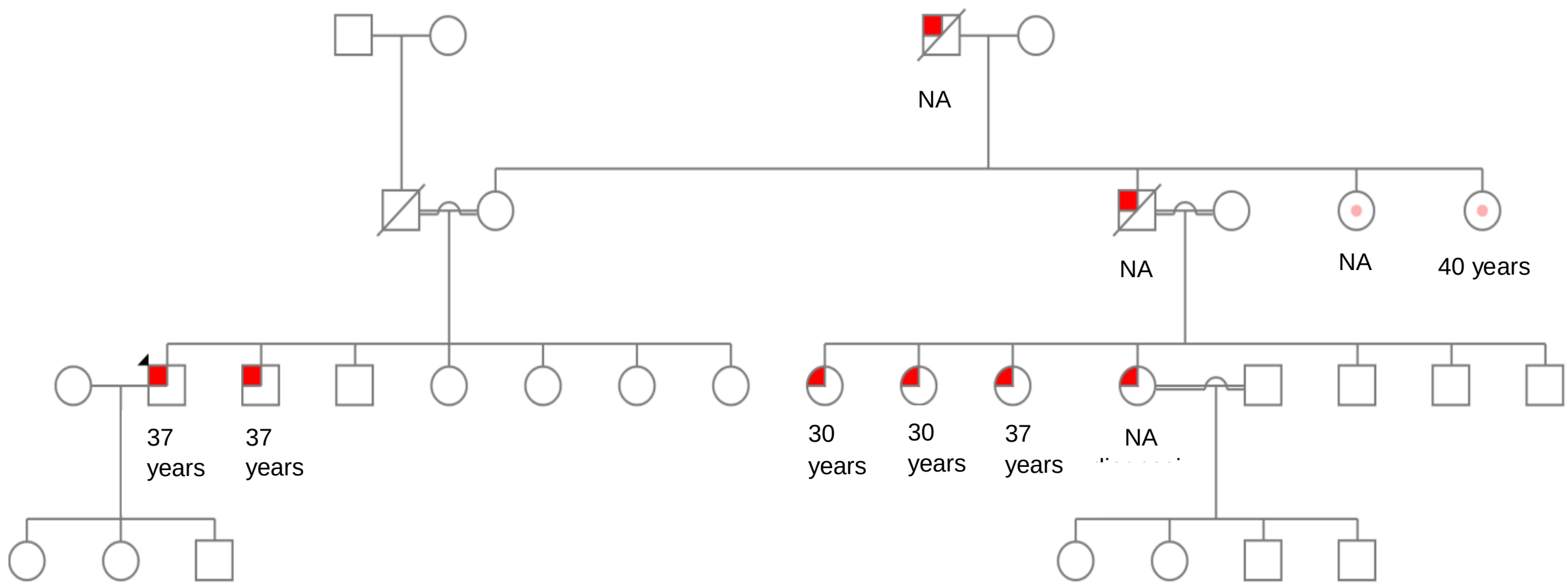

Patient #9 (140F25SD)

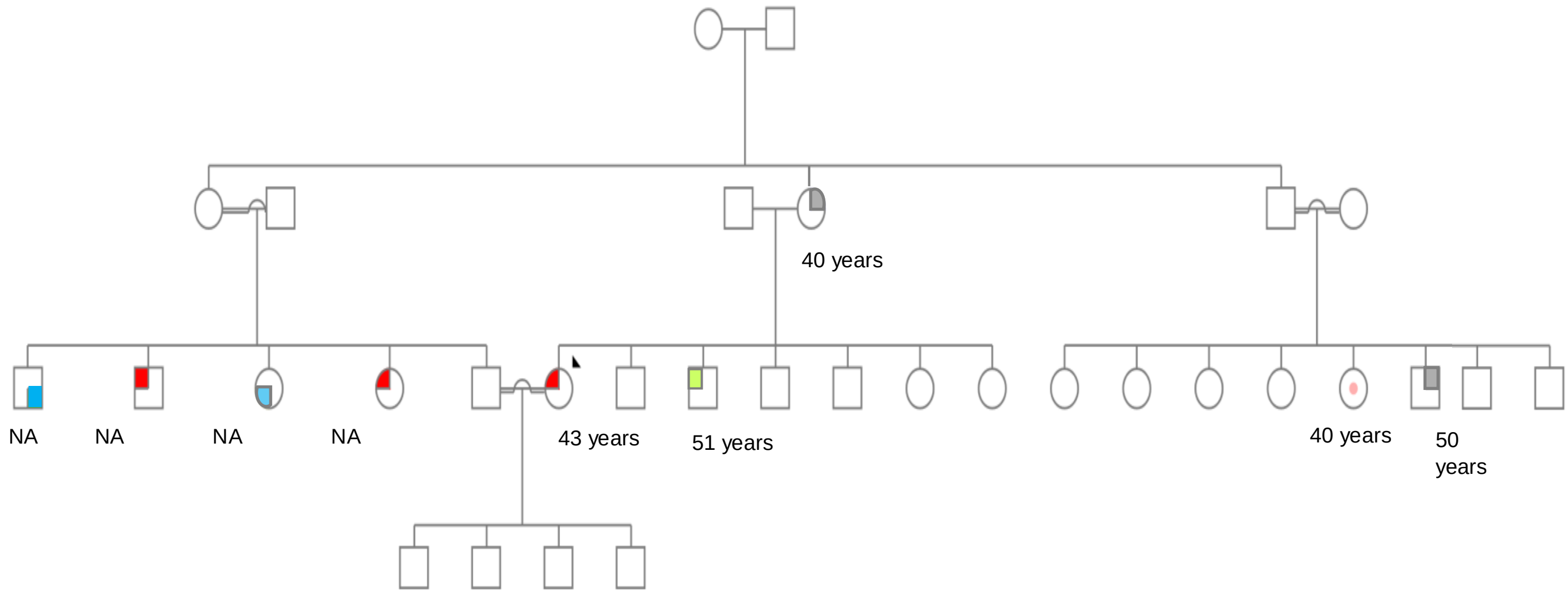

Patient #10 (140F20SD)

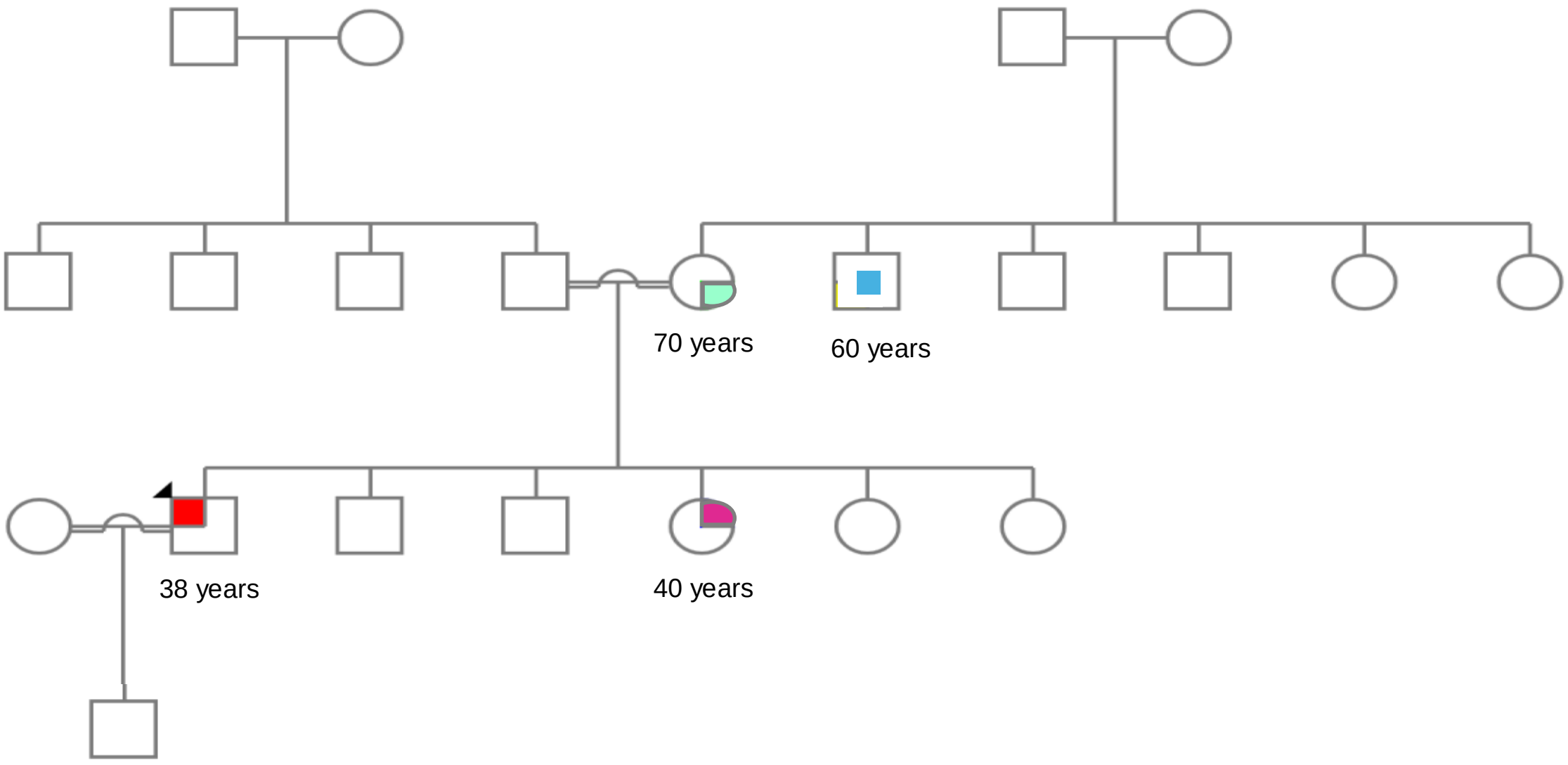

Patient #11 (140F46SD)

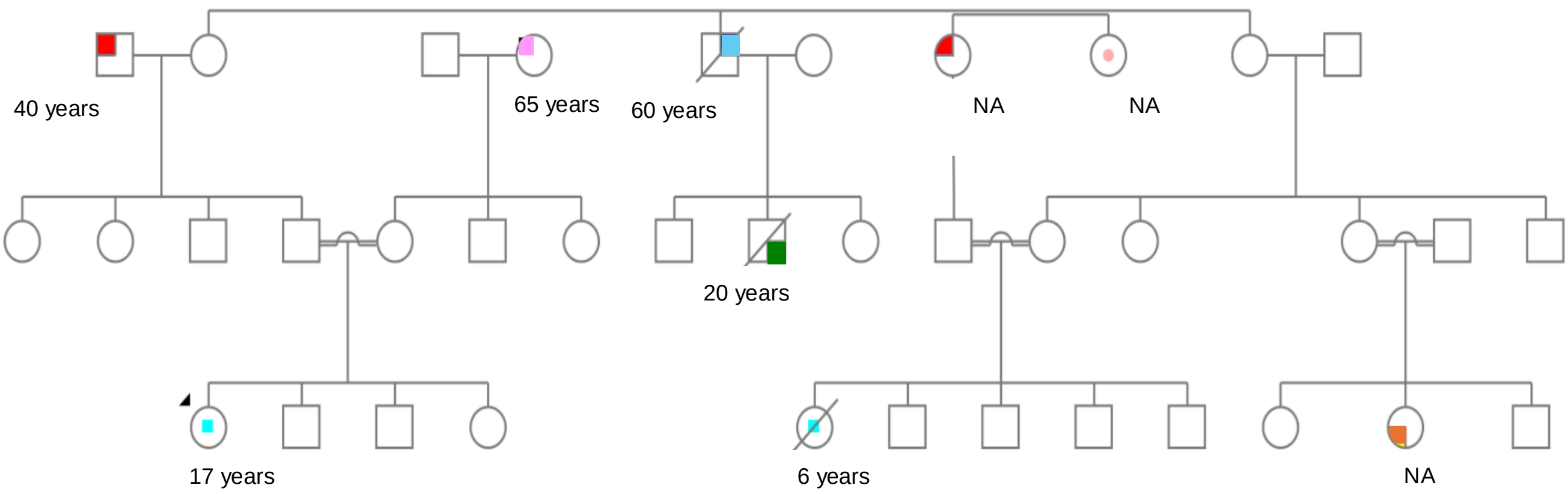

Patient #12 & 13 (140F42S1D & 140F42S2D)

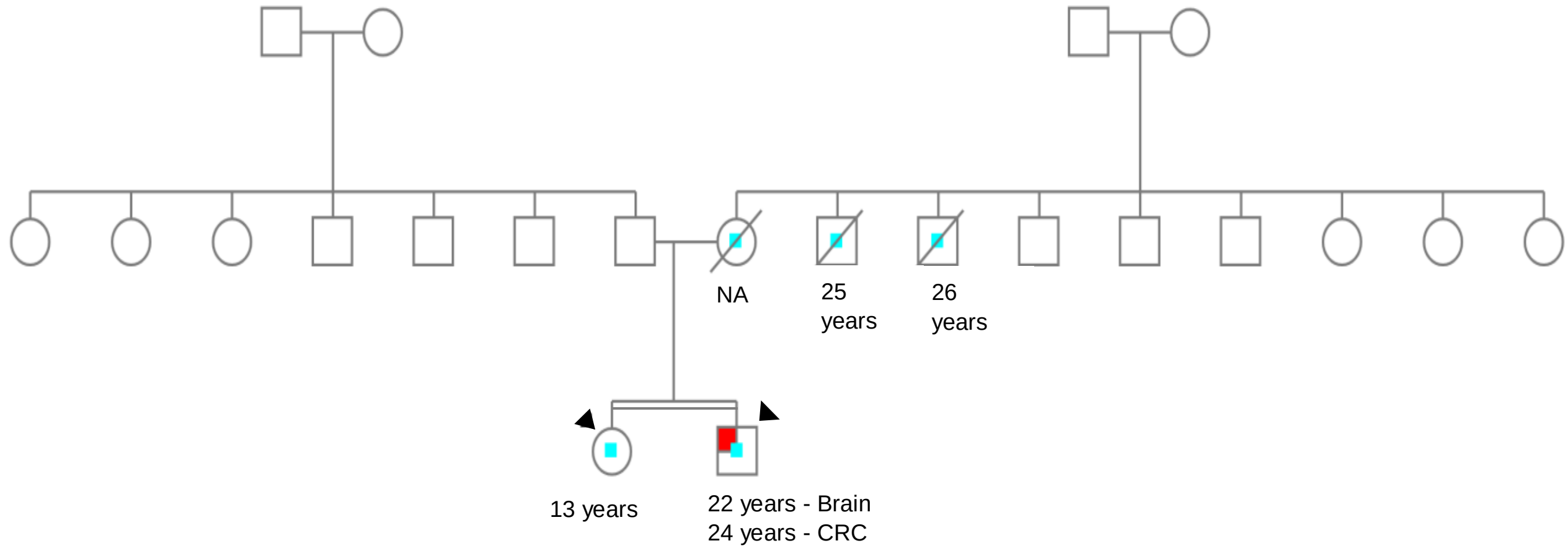

Patient #14 (140F45SD)

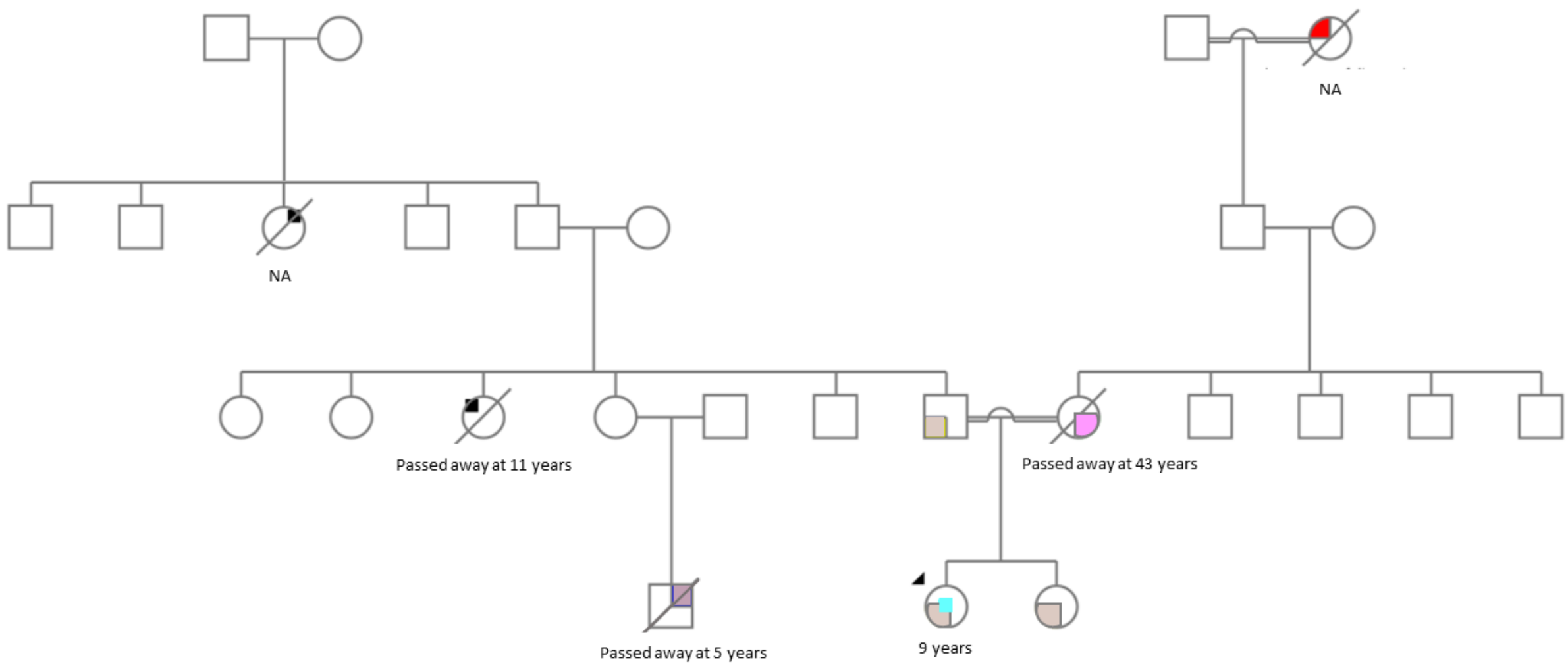

Pedigrees' Key:

- Colorectal carcinoma
- Testicular malignancy
- Gastric malignancy
- Uterine malignancy
- Prostate malignancy
- Esophageal malignancy
- Lymphoma
- Leukemia
- Parathyroid tumor
- Unspecified tongue malignancy
- Unspecified bone sarcoma
- Unspecified tumor in the jaw
- Unspecified sarcoma
- Brain tumor
- Breast cancer
- Pancreatic cancer
- Skin malignancy
- Pilomatixoma
- Liver malignancy
- Unspecified gastrointestinal tumor
- Lung cancer
- Thyroid tumor
- Keratosis
- Congenital heart disease
- Unknown malignancy
- Unspecified Gastrointestinal Tumor
