# Supplementary material for: Molecular characterization of mismatch repair deficient tumors in young Jordanian patients
Source: Discov Oncol. 2026 Aug 17;17:1198. doi: 10.1007/s12672-026-05717-3 (PMC13481598; doi:10.1007/s12672-026-05717-3)
Supplement: Supplementary file 4 [file 12672_2026_5717_MOESM2_ESM.docx]

Table 1 List of genes analyzed using the cancer predisposition sequencing (CPS) panel.

| ABCB11 | ACD | ACVR1 | AIP | AKT1 |
| --- | --- | --- | --- | --- |
| ALK | ANTXR2 | APC | ARID5B | ASXL1 |
| ATM | AXIN2 | ATRX | BAP1 | BARD1 |
| BLM | BMPR1A | BRAF | BRCA1 | BRCA2 |
| BRIP1 | BUB1B | CBL | CDC73 | CDH1 |
| CDK4 | CDKN1B | CDKN1C | CDKN2A | CEBPA |
| CHEK2 | COL7A1 | CREBBP | CTC1 | CYLD |
| DDB2 | DDX41 | DGCR8 | DHCR7 | DICER1 |
| DIS3L2 | DKC1 | DOCK8 | EGFR | ELANE |
| IKBKAP | EP300 | EPAS1 | EPCAM | ERCC1 |
| ERCC2 | ERCC3 | ERCC4 | ERCC5 | ETV6 |
| EXT1 | EXT2 | EZH2 | FAH | FANCA |
| FANCB | FANCC | FANCD2 | FANCE | FANCF |
| FANCG | FANCI | FANCL | FANCM | FAS |
| FGFR1 | FGFR2 | FGFR3 | FH | FLCN |
| FMR1 | GATA2 | GATA3 | GBA | GJB2 |
| GLI3 | GNAS | GPC3 | GPC4 | GPR101 |
| GPR161 | H19 | HAVCR2 | HFE | HMBS |
| HRAS | IDH1 | IDH2 | IKZF1 | ITK |
| KCNQ1 | KCNQ1OT1 | KIT | KLLN | KRAS |
| L2HGDH | LZTR1 | MAD2L2 | MAP2K1 | MAP2K2 |
| MAX | MEN1 | MET | MITF | MLH1 |
| MNX1 | MRAS | MRE11A | MSH2 | MSH6 |
| MTAP | MUTYH | NBN | NF1 | NF2 |
| NFIX | NHP2 | NOP10 | NPM1 | NRAS |
| NSD1 | NTRK1 | PALB2 | PAX5 | PDGFRA |
| PDGFRB | PHOX2B | PIK3CA | PINK1 | PMS2 |
| POLD1 | POLE | POLH | POT1 | PRKAR1A |
| PRSS1 | PTCH1 | PTEN | PTPN11 | RAD50 |
| RAD51 | RAD51C | RAD51D | RAF1 | RB1 |
| RECQL4 | REST | RET | RFWD3 | RHBDF2 |
| RIT1 | RMRP | RNF43 | RRAS | RRAS2 |
| RUNX1 | SAMD9 | SAMD9L | SBDS | SDHA |
| SDHAF2 | SDHB | SDHC | SDHD | SERPINA1 |
| SETBP1 | SH2B3 | SH2D1A | SHOC2 | SLC25A13 |
| SLX4 | SMAD4 | SMARCA2 | SMARCA4 | SMARCB1 |
| SMARCE1 | SMARCAL1 | SOS1 | SOS2 | SPEN |
| SPINK1 | SPRED1 | SRY | STAT3 | STK11 |
| SUFU | TERT | TFAP2A | TGFBR1 | TINF2 |
| TMEM127 | TP53 | TRIM28 | TRIM37 | TSC1 |
| TSC2 | UBE2T | UROD | VHL | WAS |
| WRAP53 | WRN | WT1 | XIAP | XPA |
| XPC | XRCC2 | XRCC3 |  |  |
