# Supplementary material for: Molecular characterization of mismatch repair deficient tumors in young Jordanian patients
Source: Discov Oncol. 2026 Aug 17;17:1198. doi: 10.1007/s12672-026-05717-3 (PMC13481598; doi:10.1007/s12672-026-05717-3)
Supplement: Supplementary file 5 [file 12672_2026_5717_MOESM5_ESM.docx]

**Mutational Signatures of Tumors Included in the Study**

**Patient #1 (140F4SD)**

**Patient #2 (140F12SD)**

**Patient #3 (140F24SD)**

**Patient #4 (140F2SD)_Primary tumor:**

**Patient #4 (140F2SD)_Relapse:**

**Patient #5 (140F30SD)**

**Patient #6 (140F29S1D)**

**Patient #7 (140F29S2D)**

**Patient #8 (140F19SD****1)_Primary tumor:**

**Patient #8 (140F19SD1)_Relapse:**

**Patient #9 (140F25SD)**

**Patient #10 (140F20SD)**

**Patient #11 (140F46SD)**

**Patient #12 (140F42S1D)**

**Patient #13 (140F42S2D)**

**Patient #14 (140F45SD)**

|  |  | Patient#1 | Patient#2 | Patient#3 | Patient#4 | Patient#5 | Patient#6 | Patient#7 | Patient#8 | Patient#9 | Patient#10 | Patient#11 | Patient#12 | Patient#13 | Patient#14 |
| --- | --- | --- | --- | --- | --- | --- | --- | --- | --- | --- | --- | --- | --- | --- | --- |
| Signature | Proposed_Etiology | 140F4SD | 140F12SD | 140F24SD | 140F2SD | 140F30SD | 140F29S1D | 140F29S2D | 140F19SD | 140F25SD | 140F20SD | 140F46SD | 140F42S1D | 140F42S2D | 140F45SD |
| SBS1 | Deamination of 5-methylcytosine | 0.32 | 0.423 | 0.052 | 0.282 | 0.15 | 0.046 | 0.125 | 0.18 | 0.18 | 0.341 | 0.767 | 0.355 | 0.085 | 0.024 |
| SBS2 | APOBEC activity |  |  |  |  | 0.059 |  |  |  |  |  |  |  |  |  |
| SBS5 | Unknown | 0.343 | 0.291 | 0.436 | 0.297 | 0.539 | 0.356 | 0.345 | 0.409 | 0.453 | 0.659 | 0.233 | 0.332 | 0.368 | 0.346 |
| SBS6 | Defective DNA mismatch repair | 0.337 | 0.227 | 0.435 | 0.283 |  |  |  |  | 0.274 |  |  |  |  |  |
| SBS7a | Ultraviolet light exposure |  |  | 0.076 |  |  |  |  |  |  |  |  |  |  |  |
| SBS10a | POLE mutation |  |  |  |  |  |  |  |  |  |  |  |  | 0.057 |  |
| SBS10b | POLE mutation |  | 0.059 |  |  |  |  |  |  |  |  |  |  | 0.091 |  |
| SBS12 | Unknown |  |  |  |  | 0.251 | 0.268 |  |  |  |  |  |  |  |  |
| SBS14 | Concurrent POLE mutation and mismatch repair deficiency |  |  |  |  |  |  |  |  |  |  |  |  | 0.103 |  |
| SBS15 | Defective DNA mismatch repair |  |  |  |  |  | 0.162 | 0.191 | 0.129 |  |  |  | 0.202 | 0.296 | 0.305 |
| SBS20 | Concurrent POLD1 mutation and mismatch repair deficiency |  |  |  |  |  |  |  |  |  |  |  | 0.112 |  |  |
| SBS26 | Defective DNA mismatch repair |  |  |  |  |  |  | 0.338 |  |  |  |  |  |  | 0.325 |
| SBS44 | Defective DNA mismatch repair |  |  |  |  |  |  |  | 0.281 |  |  |  |  |  |  |
| SBS54 | Sequencing artefact |  |  |  |  |  | 0.169 |  |  | 0.092 |  |  |  |  |  |
| SBS58 | Sequencing artefact |  |  |  | 0.139 |  |  |  |  |  |  |  |  |  |  |
